# Muhenrins A–C, pimarane-type diterpenoids from *Munronia henryi*†

**DOI:** 10.1039/d5ra04525h

**Published:** 2025-07-18

**Authors:** Wan-Bai Su, Xiao-Meng Hou, Ling Zhang, Li Yan, Zhi-Yang Tang, Yang Yu, Jin-Song Liu, Yun-Peng Sun, Guo-Kai Wang

**Affiliations:** a ^a^, School of Pharmacy, Anhui University of Chinese Medicine, Anhui Province Key Laboratory of Bioactive Natural Products Hefei 230012 P.R. China sunyp@ahtcm.edu.cn wanggk@ahtcm.edu.cn

## Abstract

Three undescribed pimarane-type diterpenoids, muhenrins A–C (1–3), along with a known analogue were isolated from the petroleum ether extract of *Munronia henryi* (Meliaceae). Their structures, including absolute configurations, were elucidated by means of various spectroscopic methods (IR, UV, HR-ESI-MS, NMR), single-crystal X-ray diffraction, ECD and NMR calculations. Muhenrin A (1) features a unique 6,7-*seco* pimarane skeleton, and its putative biosynthetic pathways have been proposed. Compounds 1–4 showed weak inhibitory activity against NO production in LPS-induced RAW 264.7 cells, with inhibition rates ranging from 13.73 – 32.35% at 50 μM concentrations.

## Abstract 2

Three pimarane-type diterpenoids, muhenrins A–C (1–3), along with a known analogue were isolated from *Munronia henryi* (Meliaceae).

## Graphical abstract

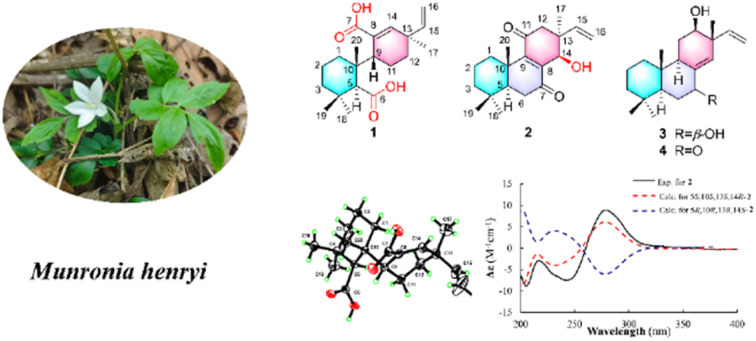

## Introduction

The plant genus *Munronia* (Meliaceae) comprises approximately 15 species, primarily distributed in China, Sri Lanka, India, Indonesia, and the Philippines. Among them, about 7–8 species native to China and is traditionally utilized in folk medicine, particularly among ethnic minorities.^1^ The whole herb of *Munronia* is widely employed to treat malaria, rheumatic joint pain, coughs, stomachaches, and has demonstrated notable insecticidal activity.^2^ Although the medicinal value of *Munronia* plants has been documented in numerous ethnic medical classics, scientific data on their chemical composition and pharmacological effects remain limited. Current studies reveal that *Munronia* species are rich in limonoids and triterpenoids,^2–5^ which exhibit notable biological activities, including anti-inflammatory,^6^ anti-tumor,^2^ anti-TMV (tobacco mosaic virus),^4^ and anti-proliferative effects.^5^ Our research group previously isolated 74 limonoids, 4 triterpenoids, and 2 pregnanes from *Munronia unifoliolata* Oliv.^6–8^ Among these, munronoid I demonstrated significant anti-inflammatory activity by markedly suppressing IL-1β release through inhibition of NLRP3 inflammasome initiation and assembly.^6^ To identify additional anti-inflammatory compounds from *Munronia* species, we performed a systematic investigation of the chemical constituents of *Munronia henryi* and screened them for anti-inflammatory activity. This study led to the isolation of three pimarane-type diterpenoids from *M. henryi*.

**Fig. 1:**
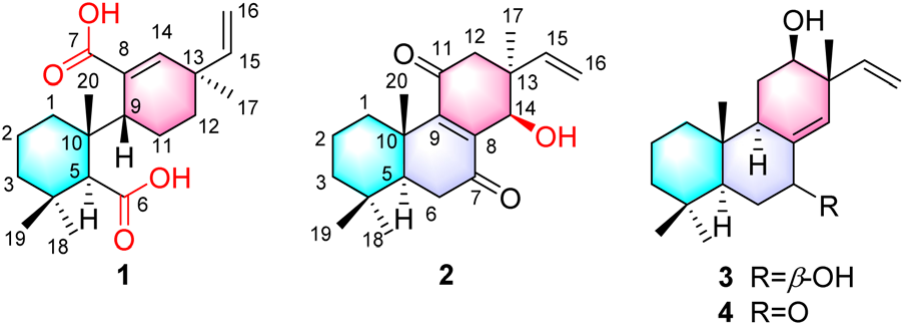
The structures of compounds 1–4.

Pimarane diterpenoids are characterized by a 4, 4, 10, 13-tetramethylperhydrophenanthrene core skeleton and are classified into pimarane, isopimrane, *ent*-pimarane and *ent*-isopimrane based on their stereochemical configurations.^9^ These compounds primarily occur in plants from the *Lamiaceae*, *Zingiberaceae*, and *Cupressaceae* families, as well as in fungi and marine organisms. To date, over 360 pimarane-type molecules have been reported, demonstrating notable cytotoxicity, anti-inflammatory, and antimicrobial activities.^10^ However, such diterpenoids are rarely documented in Meliaceae plants, with only minor quantities isolated from *Dysoxylum*, *Guarea*, and *Chukrasia* species.^11^ This study reports, for the first time, the presence of pimarane-type diterpenoids in *Munronia* plants, including compound 1, which features a unique 6,7-*seco* pimarane skeleton (Fig. 1). Additionally, their cytotoxic and anti-inflammatory activities are evaluated.

## Results and discussion

Compound 1 was obtained as a colorless square crystal, with a molecular formula of C_20_H_30_O_4_, as established by HR-ESI-MS (*m*/*z* 357.2046 [M + Na]^+^, calcd 357.2036), indicative of six degrees of unsaturation. The ^1^H NMR spectrum (Table 1) showed the presence of four olefinic protons (*δ*_H_ 6.33, 1H, s; 5.73, 1H, dd, *J* = 17.4, 10.6 Hz; 4.96, 1H, d, *J* = 10.6 Hz; 4.82, 1H, d, *J* = 17.4 Hz), and four tertiary methyl groups (*δ*_H_ 1.21, 1.16, 1.14 and 1.03, each 3H, s). Combined analysis of the ^13^C NMR and HSQC spectra (Table 1) exhibited 20 carbon signals, including four methyls, six methylenes (one olefinic at *δ*_C_ 112.7), four methines (two olefinic at *δ*_C_ 147.1, 144.7), six quaternary carbons (two carboxyls at *δ*_C_ 177.1, 174.9 and one olefinic at *δ*_C_ 135.3). The above data suggest that compound 1 contains one trisubstituted double bond, one terminal double bond, and four tertiary methyl groups, which are the characteristic fragments of pimarane-type diterpenoid.^12^ However, the six degrees of unsaturation of compound 1 (accounting for two double bonds and two carboxyl groups) imply the presence of only two rings in its structure. Additionally, the two carboxyl groups indicate that the pimarane skeleton of compound 1 has undergone ring-opening.

**Table 1:** ^1^H NMR (600 MHz) and ^13^C NMR (150 MHz) data of compounds 1–3 (*J* in Hz)

No	1^a^		2^a^		3^b^	
	*δ*_C_	*δ*_H_ (*J* in Hz)	*δ*_C_	*δ*_H_ (*J* in Hz)	*δ*_C_	*δ*_H_ (*J* in Hz)
1α	36.5	1.49, m^c^	35.0	0.96, dd (13.2, 3.7)	39.1	1.00, m
1β		1.37, m		2.69, br d (13.2)		1.66, m
2α	19.8	1.50, m^c^	18.5	1.52, m	19.0	
2β		1.62, m^c^		1.70, qt (13.7, 3.5)		1.48, m^c^
3α	42.0	1.19, m^c^	40.9	1.20, dd (13.6, 3.9)	41.9	1.17, td (13.3,4.5)
3β		1.53, m^c^		1.46, br d (13.4)		1.45, m^c^
4	34.5		33.1		33.3	
5	58.6	2.55, s	50.1	1.59, dd (15.0, 3.2)	52.0	3.87, t (8.0)
6α	177.1		35.7	2.56, dd (17.8, 3.2)	32.6	2.00, m
6β				2.44, dd (17.8, 15.0)		1.29, m^c^
7	174.9		204.1		72.5	3.99, br s
8	135.3		141.1		139.4	
9	41.6	2.78, br s	156.0		50.3	1.93, m
10	42.7		39.4		38.4	
11α	21.5	1.60, m^c^	200.3		26.3	1.80, m
11β		2.06, br d (14.7)				1.53, m
12α	33.8	1.54, m^c^	45.7	2.53, d (17.0)	73.2	3.57, dd (12.3, 3.9)
12β		1.74, td (13.0, 3.0)		2.65, d (17.0)		
13	38.9		41.7		42.7	
14	144.7	6.33, s	68.9	4.55, d (2.6)	124.9	
15	147.1	5.73, dd (17.4, 10.6)	143.9	5.75, dd (17.6, 10.9)	146.2	5.80, dd (17.4, 10.8)
16a	112.7	4.82, d (17.6)	113.9	4.93, d (17.6)	114.1	5.14, d (17.4)
16b		4.96, d (10.6)		5.04, d (10.9)		5.15, d (10.8)
17	27.2	1.14, s	22.4	1.16, s	17.5	1.08, s
18	34.7	1.03, s	33.0	0.89, s	33.8	0.91, s
19	24.5	1.16, s	21.4	0.93, s	22.2	0.87, s
20	22.5	1.21, s	17.3	1.32, s	14.8	0.82, s

a ^1^H NMR (500 MHz) and ^13^C NMR (125 MHz) data in CD_3_OD.

b ^1^H NMR (600 MHz) and ^13^C NMR (150 MHz) data in CDCl_3_.

c overlapped.

**Fig. 2:**
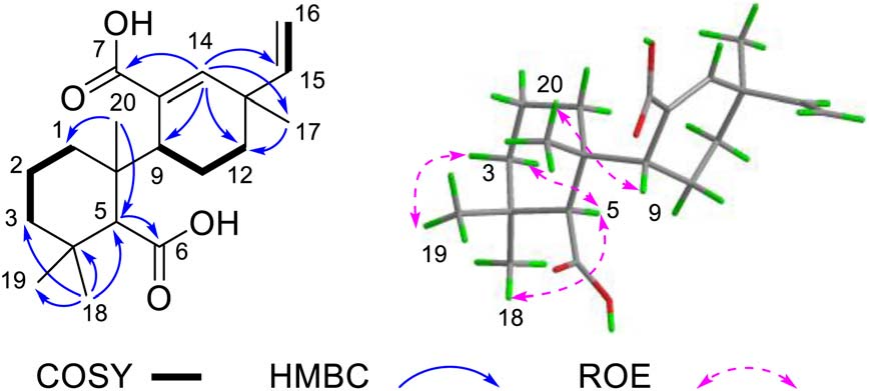
Key 2D NMR correlations of 1.

The 2D NMR spectra revealed key structural features of compound 1 (Fig. 2). In the HMBC spectrum, H_3_-18 (*δ*_H_ 1.03, 3H, s) showed correlations to C-19 (*δ*_C_ 24.5), C-3 (*δ*_C_ 42.0), C-4 (*δ*_C_ 34.5), and C-5 (*δ*_C_ 58.6). These correlations, together with the ^1^H–^1^H COSY cross-peaks between H-1/H-2/H-3, confirmed the intact nature of ring A. Furthermore, HMBC correlations of H_3_-17 (*δ*_H_ 1.14, s) to C-12 (*δ*_C_ 33.8) and C-14 (*δ*_C_ 144.7), along with those of H-14 (*δ*_H_ 6.33, s) to C-9 (*δ*_C_ 41.6) and C-12, combined with the ^1^H–^1^H COSY cross-peaks of H-9/H-11/H-12, established the complete ring C structure. Furthermore, the HMBC correlations between H-14 and *δ*_C_ 174.9, and between H-5 and *δ*_C_ 177.1 suggested that the two carboxyl groups belong to C-7 and C-6, respectively. This assignment was further supported by the molecular formula derived from HR-MS analysis. Consequently, compound 1 was identified as a 6,7-*seco* pimarane-type diterpenoid, as illustrated in the Fig. 1. Notably, the ^13^C NMR spectrum of 1 exhibits signal broadening, likely attributable to steric hindrance from the carboxyl group restricting free rotation about the C9–C10 single bond.^13^

**Fig. 3:**
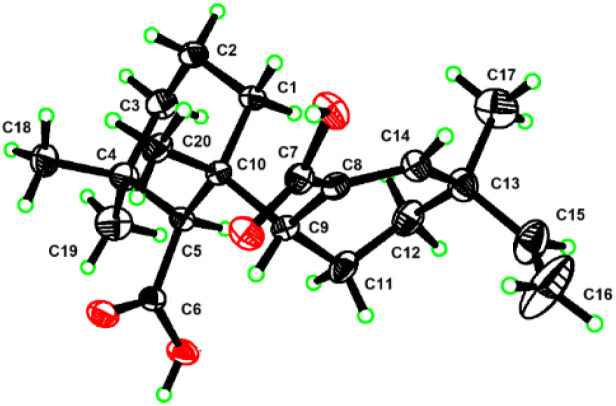
X-ray structure of 1.

The relative configuration of compound 1 was partially established through ROESY correlations (Fig. 2). The cross-peaks between H_3_-18/H-5/H-3α indicated α-orientation for both H_3_-18 and H-5, while the correlation between H_3_-19 and H-3β supported β-orientation for H_3_-19. However, the stereoconfiguration of compound 1 could not be determined by ROESY due to the free rotation about the C9–C10 single bond. The absolute configuration was ultimately determined by X-ray crystallographic analysis of single crystals (Fig. 3) obtained from methanol solution, which established the 5*S*, 9*R*, 10*R*, 13*R* configuration [Flack parameter = −0.06(13)].

**Fig. 4:**
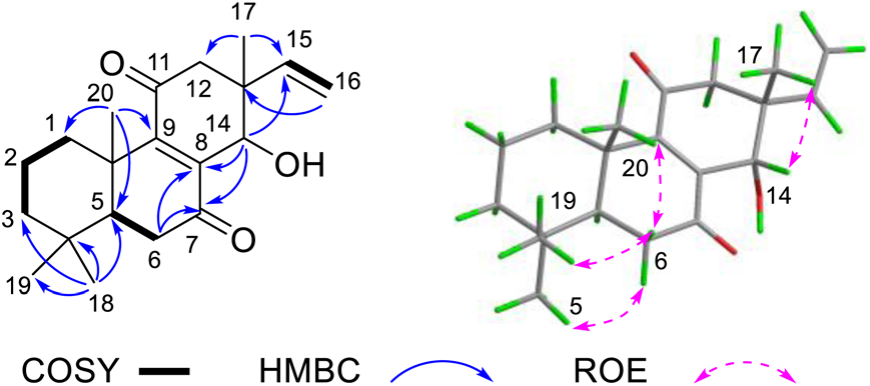
Key 2D NMR correlations of 2.

Compound 2 was obtained as a white powder. Its molecular formula was established as C_20_H_28_O_3_ by HR-ESI-MS (*m*/*z* 315.1960 [M − H]^−^), corresponding to seven degrees of unsaturation. The ^1^H NMR spectrum of 2 (Table 1) displayed signals characteristic of a terminal double bond (*δ*_H_ 5.75, dd, *J* = 17.6, 10.9 Hz; 5.04, d, *J* = 10.9 Hz; 4.93, d, *J* = 17.6 Hz) and four tertiary methyl groups (*δ*_H_ 1.32, 1.16, 0.93 and 0.89, each 3H, s). The ^13^C spectrum of 2, combined with the HSQC spectrum, revealed the presence of seven quaternary carbons (including two keto carbonyls at *δ*_C_ 204.1 and 200.3, and two olefinic carbons at *δ*_C_ 156.0 and 141.4), three methine carbons (one olefinic at *δ*_C_ 143.9, and one oxygenated at *δ*_C_ 69.0), six methylene carbons (one olefinic at *δ*_C_ 113.9) and four methyl carbons. These signals account for four degrees of unsaturation, while the remaining three unsaturations suggest that 2 is a tricyclic diterpenoid. The spin systems identified in the ^1^H–^1^H COSY spectrum (Fig. 4), specifically H-1/H-2/H-3 and H-5/H-6, along with key HMBC correlations (H_3_-18 to C-3/C-4/C-5; H-20 to C-1/C-5/C-9/C-10; and H-6 to C-7/C-8/C-10), were crucial for establishing the structures of rings A and B. Combined with the terminal double bond feature and HMBC correlations from H-17 to C-12, C-14, C-15, compound 2 was identified as a pimarane-type diterpenoid, exhibiting a planar structure similar to known compound 12β-Hydroxy-7,11-dioxopimal-8,15-dien.^14^ The sole structural difference involves the position of hydroxyl group substitution in 2, the hydroxyl group is located at C-14, whereas in 12β-hydroxy-7,11-dioxopimar-8,15-dien, it is positioned at C-12. This distinction was confirmed by the HMBC correlations observed between H-14 and C-7/C-8.

**Fig. 5:**
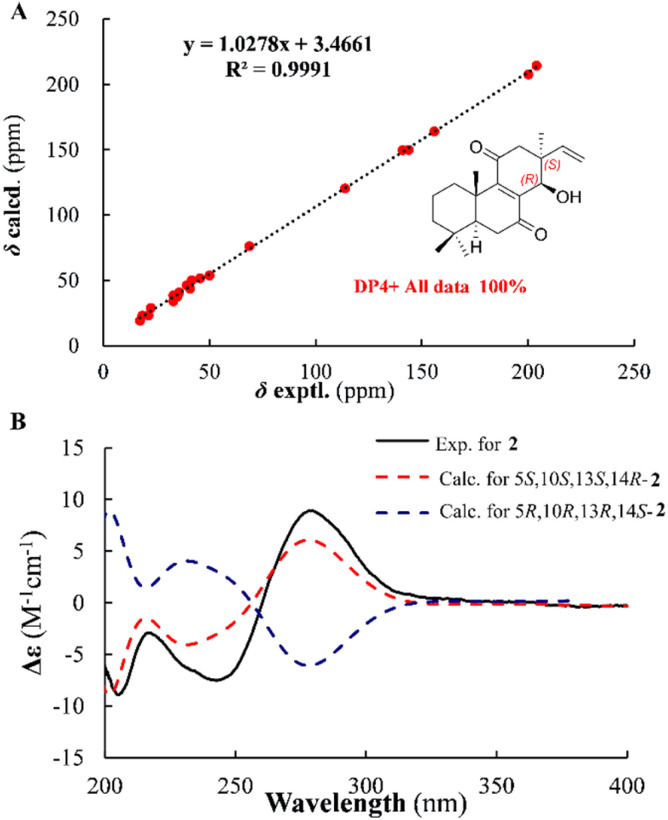
Correlations between experimental and calculated ^13^C NMR chemical shifts of (13S*, 14R*)-2 (A) and ECD calculations for (13*S*, 14*R*)-2 (B).

The ROESY spectrum revealed key spatial relationships: correlations between H_3_-19 (*δ*_H_ 0.93), H-6β (*δ*_H_ 2.44), H_3_-20 (*δ*_H_ 2.69) indicated their cofacial arrangement and β-orientation. Similarly, the H_3_-18 (*δ*_H_ 0.89)/H-6α (*δ*_H_ 2.56) correlation suggested their cofacial α-orientation (Fig. 4). The α-orientation of H-5 was further supported by its small coupling constant (*J*_5,6α_ = 3.2 Hz). While the ROESY correlation between H_3_-17(*δ*_H_ 1.16) and H-14 (*δ*_H_ 4.55) confirmed their cofacial arrangement, two possible relative configurations (13S*, 14R* and 13R*, 14S*) remained. To resolve this ambiguity, we calculated the ^13^C chemical shifts for both epimers (Fig. 5A). DP4+ analysis of both ^1^H and ^13^C NMR data unequivocally identified (13S*, 14R*)-2 as the correct structure (100% probability, Fig. S3†).^15,16^ The absolute configuration (5*S*, 10*S*, 13*S*, 14*R*) was ultimately confirmed by excellent agreement between experimental and calculated ECD spectra (Fig. 5B).

**Fig. 6:**
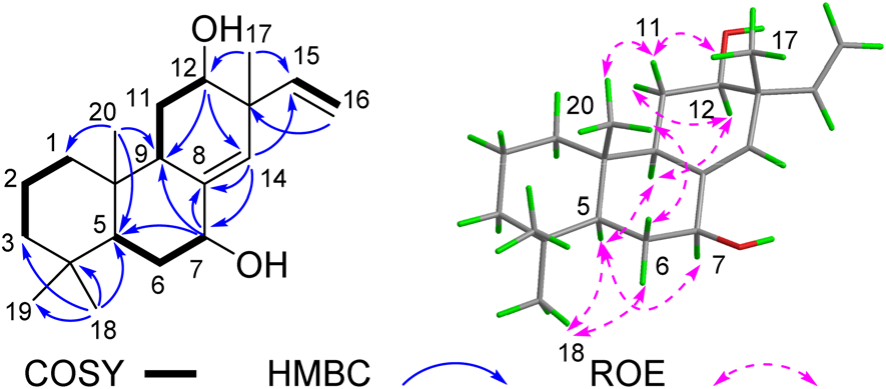
Key 2D NMR correlations of 3.

Compound 3 was isolated as a white powder. Its molecular formula (C_20_H_32_O_2_) was established by ^13^C NMR and HR-ESI-MS (observed [M − H]^−^ at *m/z* 327.2295), implying five degrees of unsaturation. The ^1^H NMR spectrum (Table 1) displayed characteristic methyl group signals (*δ*_H_ 1.08, 0.91, 0.87, 0.82, each, 3H, s) and olefinic proton signals (*δ*_H_ 5.80, dd, *J* = 17.4, 10.8 Hz; 5.54, d, *J* = 2.0 Hz; 5.15, d, *J* = 10.8 Hz; 5.14, d, *J* = 17.4 Hz), suggesting a pimarane-type diterpenoid skeleton analogous to compound 2. Comparative analysis of 1D and 2D NMR data revealed close structural similarity to (5*S*, 9*R*, 10*S*, 12*R*, 13*R*)-12-hydroxyisopimara-8(14),15-dien-7-one,^17^ with only differences of the absence of a carbonyl carbon and the presence of an additional oxygenated carbon. The observed mass difference of 2 suggested reduction of the C-7 carbonyl group to a hydroxyl group in compound 3. This structural modification was confirmed by HMBC correlations from H-7 (*δ*_H_ 3.99) to C-5 (*δ*_C_ 52.0), C-8 (*δ*_C_ 139.4), C-9 (*δ*_C_ 50.3), along with ^1^H–^1^H COSY cross-peaks between H-5/H-6/H-7 (Fig. 6).

ROESY correlations observed among H_3_-18/H-5/H-9/H-12, H-5/H-7, and H_3_-18/H-6α (*δ*_H_ 2.00) established their cofacial arrangement and α-orientation. Complementary ROESY cross-peaks between H_3_-17/H-11β (*δ*_H_ 1.53)/H_3_-20/H-6β (*δ*_H_ 1.29) confirmed their β-orientation. The absolute configuration of 3 was determined to be 5*S*, 7*S*, 9*R*, 10*S*, 12*R*, 13*R* through comparison of experimental and calculated ECD spectra (Fig. S2†).

In addition to the aforementioned compounds, a known pimarane-type diterpenoid was isolated from *M. henryi.* Based on comparison of its spectroscopic data with literature values,^17^ this compound was identified as (5*S*, 9*R*, 10*S*, 12*R*, 13*R*)-12-hydroxyisopimara-8(14),15-dien-7-one (4).

According to previous literature, pimarane-type diterpenoids have demonstrated significant cytotoxic and anti-inflammatory potential.^18–20^ Initially, all compounds were tested for anti-inflammatory activity using L-NMMA (50 μM) as a positive control (52.75 ± 1.28% inhibition). At 50 μM concentration, compounds 1–4 exhibited no significant inhibitory effects on NO production in LPS-induced RAW 264.7 macrophages, showing inhibition rates ranging from 13.73% to 32.35%.

Subsequently, the cytotoxicity of these compounds was evaluated against human colon cancer cells (HCT-166) and human liver cancer cells (Hep3B). None of the compounds exhibited significant cytotoxicity against either cancer cell line.

## Conclusions

In summary, four pimarane-type diterpenoids were isolated from the petroleum ether extract of *Munronia henryi*, including three previously undescribed compounds (1–3) and one known analogue. Muhenrin A (1) features a novel 6,7-*seco* pimarane skeleton, likely biosynthesized from 7α-hydroxyisopimara-8(14),15-diene.^21^ Two possible pathways are proposed for its formation (Scheme S1†). Path a: the 7-hydroxyl group of 7α-hydroxyisopimara-8(14),15-diene is oxidized to a ketone carbonyl (i), followed by Baeyer–Villiger oxidation to yield the lactone intermediate (ii).^22^ Subsequent hydrolysis and continuous oxidation produce 1. Path b: 7α-hydroxyisopimara-8(14),15-diene undergoes dehydration to form the alkene intermediate (iv), which is then directly oxidized to 1*via* dioxygenase activity.^23^ To our knowledge, this represents the first report of pimarane-type diterpenoids from *Munronia* species.

The initial biological activity assays did not yield significant results. Future studies should consider alternative cell models or explore additional biological activities, such as antibacterial or antiviral effects.^24,25^

## Experimental

### General experimental procedures

Optical rotations were measured with a P-2000 digital polarimeter (solvent: MeOH, JASCO, Japan). UV spectra were obtained on a UV-2401A spectrophotometer (solvent: MeOH, Shimadzu, Japan). Circular dichroism (CD) spectra were obtained on a JASCO(J-1500) CD spectrometer in MeOH (Applied Photophysics, Japan). HRMS-ESI was performed on an Thermo Orbitrap Exploris 120 mass spectrometer (Thermo Fisher Scientific, USA, Xevo G2 - XSQT of mass spectrometer from Waters, USA). In addition, 1D and 2D NMR spectra were recorded on Bruker DRX-500 and 600 spectrometers (Bruker, Germany) at 298 K (internal standard: TMS, solvent: CD_3_OD and CDCl_3_). Silica gel (200–300 mesh, Qingdao Haiyang, China), Sephadex LH-20 (GE, USA), and RP-18 (5 μm, Fuji Silysia Chemical, Japan) were used for column chromatography. MPLC was performed on a Büchi Sepacore System equipped with a pump manager (Büchi, Switzerland) and prep-HPLC was performed on a 1260 instrument (Agilent, USA).

### Plant material

The whole plant of *Munronia henryi* was collected in November 2022 from Wenshan Zhuang and Miao Autonomous Prefecture, Yunnan province, P. R. China, and identified by Professor Qing-Shan Yang of Anhui University of Chinese Medicine. A voucher specimen (no. 20221101) was deposited in the Department of Traditional Chinese Medicine and Natural Medicinal Chemistry at Anhui University of Chinese Medicine.

### Extraction and isolation

The dried and pulverized aerial parts of *Munronia henryi* (20 kg) were exhaustively extracted with 95% ethanol (4 × 3 h, reflux). The combined ethanolic extracts were concentrated under reduced pressure to yield a crude extract, which was subsequently suspended in H_2_O and partitioned with dichloromethane (DCM). The DCM portion was suspended in water and then extracted with petroleum ether (PE).

The PE fraction (363 g) was fractionated by silica gel column chromatography using a stepwise gradient of PE-EtOAc (100 : 0 → 0 : 100, v/v) to yield eight fractions (A–H). Fraction F (27.1 g) was further purified by ODS column chromatography with a MeOH–H_2_O gradient (40 : 60 → 100 : 0, v/v), yielding six subfractions (Fa–Ff). Subfraction Fc was chromatographed on ODS with isocratic elution (MeCN–H_2_O, 45 : 55, v/v) to afford nine fractions (Fca–Fci). Final purification of Fcc by semi-preparative HPLC (MeCN–H_2_O, 65 : 35, 8.0 mL min^−1^) yielded compound 4 (4.81 mg, *t*_R_ = 36 min).

Similarly, subfraction Fb was separated by ODS chromatography (isocratic MeCN–H_2_O, 45 : 55) into thirteen fractions (Fba–Fbn). Fraction Fbk was purified by semi-preparative HPLC (MeCN– H_2_O 57 : 43, 8.0 mL min^−1^) to give Fbka (4.71 mg, *t*_R_ = 43 min), which was further purified (MeOH–H_2_O, 75 : 25, 8.0 mL min^−1^) to afford compound 3 (2.58 mg, *t*_R_ = 28 min). Fraction Fbn was processed similarly (MeCN–H_2_O, 67 : 33 → MeOH–H_2_O, 55 : 45) to yield compound 2 (2.55 mg, *t*_R_ = 32 min) *via* intermediate fraction Fbna (5.31 mg, *t*_R_ = 45 min).

Fraction H (24.2 g) was fractionated by MCI gel column chromatography using a MeOH–H_2_O gradient (20 : 80 → 100 : 00, v/v), yielding six subfractions (Ha–Hf). Subfraction Hf was further purified by Sephadex LH-20 column chromatography (MeOH) to afford four fractions (Hfa–Hfd). Final purification of Hfb by semi-preparative HPLC (MeCN–H_2_O gradient: 0–30 min, 55 : 45; 30–35 min, 55 : 45 → 85 : 15; 35–50 min, 85 : 15; flow rate 8.0 mL min^−1^) yielded compound 1 (6.75 mg, *t*_R_ = 43 min).

#### Muhenrin A (1)

Colorless square crystal; [α]25 D − 53.0 (*c* = 0.2, MeOH); UV *λ*_max_ (MeOH) nm (log *ε*): 210 (4.62); ECD (MeOH) *λ*_max_ (Δ*ε*) 237.5(+16.72) nm; IR (KBr) cm^−1^ 3457, 2951, 2864, 2831, 1701, 1604, 1448, 1365, 1227, 1205, 1152; ^1^H and ^13^C NMR data see Table 1; HRESI-MS *m*/*z* 357.2046[M + Na]^+^ (calcd for 357.2036, C_20_H_30_O_4_Na).

#### Muhenrin B (2)

White powder; [α]25 D − 2.9 (c 0.31, MeOH); UV (MeOH) *λ*_max_ (log *ε*) 239 (2.24) nm; ECD (MeOH) *λ*_max_ (Δ*ε*) 205(−8.87) nm, 217 (−2.92) nm, 242 (−7.5) nm, 279 (+8.9) nm; IR (KBr) cm^−1^ 3394, 2922, 2831, 2716, 1600,1364, 1154, 1068, 971,775; ^1^H and ^13^C NMR data see Table 1; HR-ESI-MS *m*/*z* 315.1960 [M − H]^−^ (calcd for 315.1955, C_20_H_27_O_3_).

#### Muhenrin C (3)

White power; [α]25 D + 10.3 (c 0.13, MeOH); UV (MeOH) *λ*_max_ (log *ε*) 210 (3.12) nm; ECD (MeOH) *λ*_max_ (Δ*ε*) 299 (+0.48) nm; IR (KBr) cm^−1^ 3394, 2922, 2831, 2716, 1600,1364, 1154, 1068, 971,775; ^1^H and ^13^C NMR data see Table 1; HR-ESI-MS *m*/*z* 327.2295[M + Na]^+^(calcd for 327.2294, C_20_H_32_O_2_Na).

#### Crystal data for muhenrin A (1)

C_20_H_30_O_4_, *M* = 334.44, *a* = 12.0825(8) Å, *b* = 12.7004(8) Å, *c* = 12.9052(8) Å, *α* = 90°, *β* = 90°, *γ* = 90°, *V* = 1980.3(2) Å^3^, *T* = 170.0 K, space group *P*2_1_, *Z* = 4, μ(Cu Kα) = 0.613 mm^−1^, 17 312 reflections measured, 3988 independent reflections (*R*_int_ = 0.0736). The final *R*_1_ values were 0.0582 (*I* > 2*σ*(*I*)). The final *wR*(*F*^2^) values were 0.1559 (*I* > 2*σ*(*I*)). The final *R*_1_ values were 0.0621 (all data). The final *wR*(*F*^2^) values were 0.1622 (all data). The goodness of fit on *F*^2^ was 1.019. Flack parameter = −0.06(13). A suitable crystal was selected and recorded with a diffractometer using Cu Kα radiation. The structure was solved with the ShelXT structure solution program using direct methods and refined with the ShelXL refinement package using least squares minimisation based on Olex2 software. The crystallographic data of compounds have been deposited at the Cambridge Crystallographic Data Center with the deposition number CCDC 2452064 (1).

## Conflicts of interest

There are no conflicts to declare.

## Supplementary Material

RA-015-D5RA04525H-s001

RA-015-D5RA04525H-s002

## Data Availability

The data supporting this article have been included as part of the ESI.† Crystallographic data for muhenrin A (1) has been deposited at the CCDC under 2452064 and can be obtained from https://www.ccdc.cam.ac.uk/
