# Supplementary material for: Muhenrins A–C, pimarane-type diterpenoids from Munronia henryi
Source: RSC Adv. 2025 Jul 18;15(31):25542–7. doi: 10.1039/d5ra04525h (PMC12272833; doi:10.1039/d5ra04525h)
Supplement: RA-015-D5RA04525H-s001 [file RA-015-D5RA04525H-s001.pdf]

Supplementary Information (SI) for RSC Advances.
This journal is © The Royal Society of Chemistry 2025

Supporting Information

Muhenrins A-C, pimarane-type diterpenoids from *Munronia henryi*

Wan-Bai Su, Xiao-Meng Hou, Ling Zhang, Li Yan, Zhi-Yang Tang, Yang Yu, Jin-Song Liu, Yun-Peng Sun*, Guo-Kai Wang*

School of Pharmacy, Anhui University of Chinese Medicine, Anhui Province Key Laboratory of Bioactive Natural Products, Hefei 230012, P.R. China

*Corresponding authors.

Tel./fax: +86 551 68129167 (Y.-P. Sun); +86 551 68129123 (G.-K. Wang).

E-mail addresses: sunyp@ahtcm.edu.cn (Y.-P. Sun); wanggk@ahtcm.edu.cn (G.-K. Wang)

Table of Contents

1. Supplementary Figures 3
2. Biological assays 6
3. Computational details 8
4. Original spectra of compounds 12

NMR, MS, UV and CD spectra of compound 1 12

NMR, MS, UV and CD spectra of compound 2 17

NMR, MS, UV and CD spectra of compound 3 22

NMR spectra of compound 4 27

1. Supplementary Figures

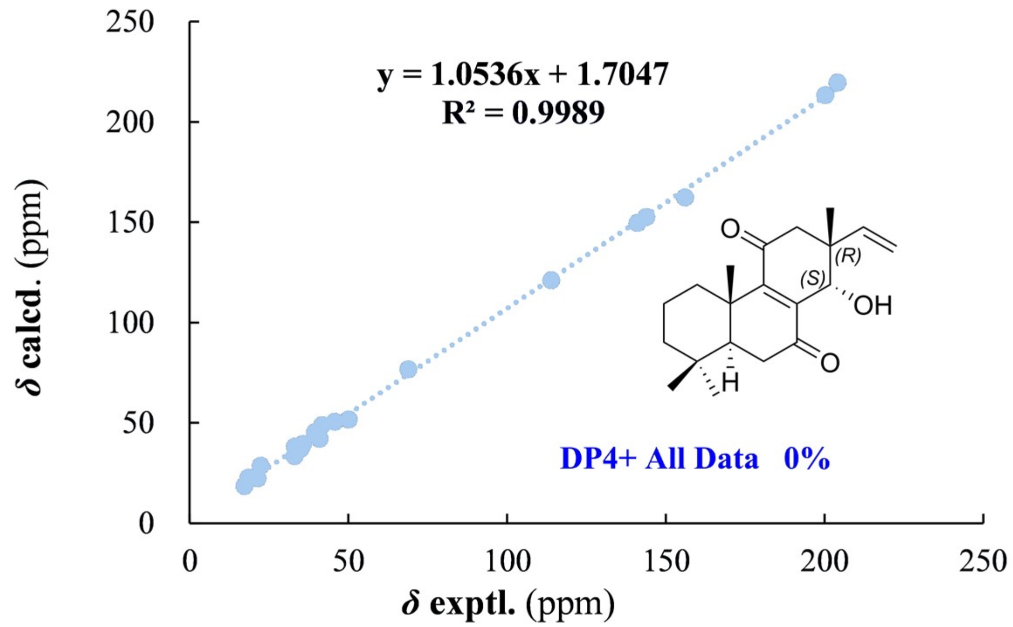

Fig. S1 Correlations between experimental and calculated ^{13}C NMR chemical shifts of (13*R**14*S**)-**2**.

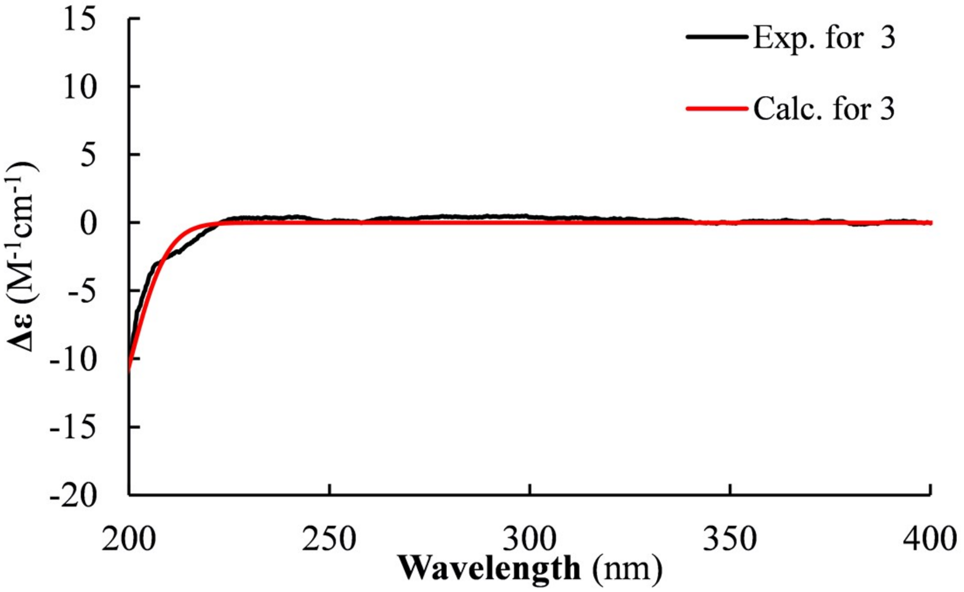

Fig. S2 Experimental and computational ECD spectra of muhenrin C (**3**)

Functional		Solvent?		Basis Set	
mPW1PW91		PCM		6-311+G(d,p)	
		DP4+	100.00%	0.00%	-
Nuclei	sp2?	xperimenta	Isomer 1	Isomer 2	Isomer 3
C		204.1	214.278461	219.658488	
C		200.3	207.658743	213.369706	
C	x	156	163.959009	162.374688	
C	x	143.9	149.923425	152.572275	
C	x	141.1	149.643724	149.635983	
C	x	113.9	120.411304	121.182363	
C		68.9	76.2938244	76.6649672	
C		50.1	53.7022368	51.7685507	
C		45.7	51.724816	50.5819701	
C		41.7	50.1242493	48.8798326	
C		40.9	43.568062	42.1387737	
C		39.4	46.1809895	45.2270843	
C		35.7	40.7902036	39.5351387	
C		35	37.4758471	37.1026045	
C		33.1	38.447864	38.1974317	
C		33	34.0368033	22.4285772	
C		22.4	28.8170355	28.651763	
C		21.4	23.1578525	33.5353672	
C		18.5	23.0196194	22.8336304	
C		17.3	19.1192515	18.4893662	
H	x	5.75	6.76226108	6.68017171	
H		5.3	4.67993918	5.39278012	
H	x	5.02	5.52930672	5.52205902	
H	x	4.93	5.42217396	5.05052283	
H		4.55	4.81375145	4.87291408	
H		2.69	3.00427695	3.01300636	
H		2.65	2.98964399	2.91540984	
H		2.53	2.51062494	2.26555935	
H		2.52	2.66584444	2.54970782	
H		2.44	2.67961194	2.47646049	
H		1.7	1.92585994	1.76742407	
H		1.59	1.79059287	1.84418972	
H		1.52	1.61264296	1.48019307	
H		1.46	1.56138945	1.40798243	
H		1.32	1.74807873	1.639513	
H		1.32	1.25513574	0.8817251	
H		1.32	1.57845473	1.30086683	
H		1.2	1.34151412	1.2529491	
H		1.16	1.27570993	1.03135857	
H		1.16	1.02898284	1.97130621	
H		1.16	1.90150168	0.89586036	
H		0.96	1.06989813	1.0427292	
H		0.93	1.60729487	0.89152724	
H		0.93	1.02538047	1.39809187	
H		0.93	0.72072004	0.57892817	
H		0.89	1.41182612	1.17390027	
H		0.89	0.67844408	0.53711249	
H		0.89	0.94731604	0.81667197	

Fig. S3 DP4+ analyses of calculated and experimental NMR chemical shifts of **2**. Isomer 1: (5*S*, 10*S*, 13*S*, 14*R*)-**2**; Isomer 2: (5*S*, 10*S*, 13*R*, 14*S*)-**2**.

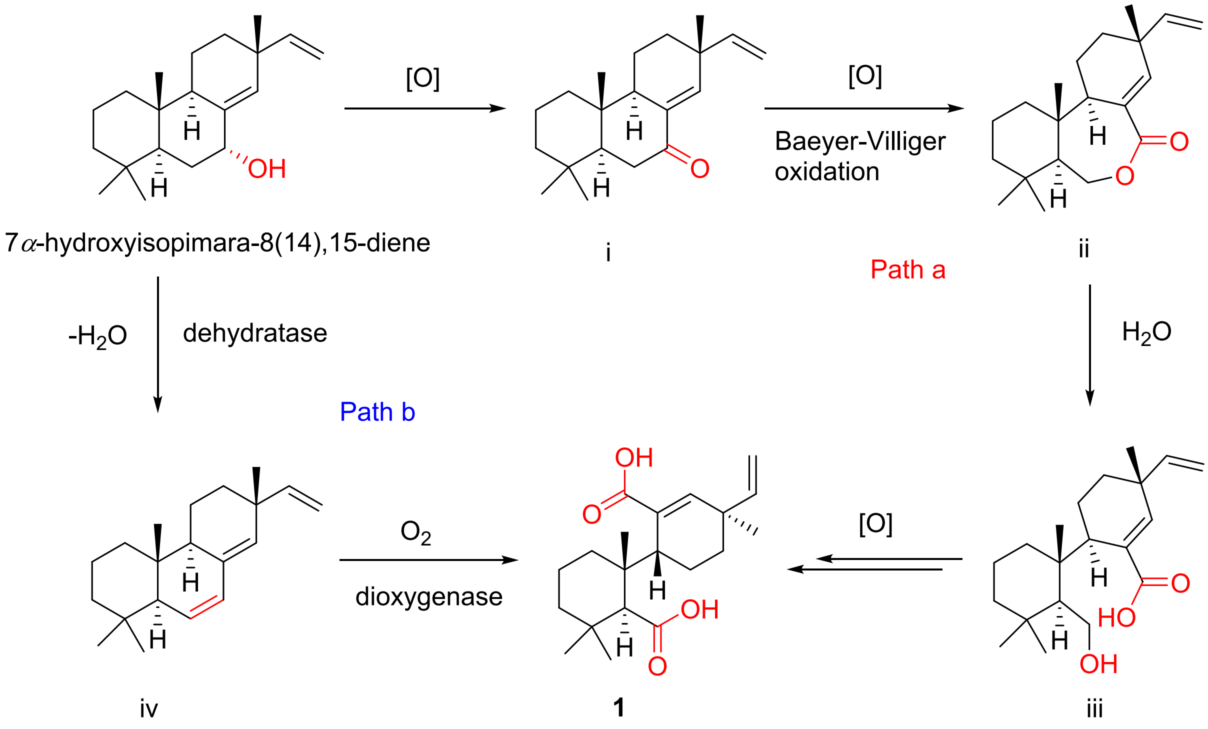

Scheme S1: Proposed biogenetic pathway of **1**.

2. Biological assays

2.1 Materials and Instrument

RAW 264.7 macrophages (Mouse Mononuclear Macrophages cells, Cat#: BFN607200597) were purchased from the Cell Bank of Chinese Sciences Academy (Shanghai, China), Human colon cancer HCT-116 cells (Cat#: CL-0096) were purchased from Procell (Wuhan, China) and human liver cancer cells (Hep3B, Cat#: CL-0102) were purchased from Procell (Wuhan, China). Dulbecco's modified eagle medium with high glucose (DMEM, Cat#: CA0002) was sourced from SparkJade (Shandong, China), and fetal bovine serum (FBS, Cat#: BL205A), 1 × phosphate buffered saline (1 × PBS, Cat#: BL302A), penicillin (1000 U/ml)-streptomycin (10 mg/ml) (Cat#: BL505A), trypsin (0.25%, Cat#: BL501A) was obtained from Biosharp (Anhui, China). 96-well tissue culture plates (Cat#:21824077E) from LABSELECT (Anhui, China), and MTT Cell Proliferation and Cytotoxicity Assay Kit (Cat#: C0009M) was from Beyotime (Shanghai, China). Automatic Microplate Reader was sourced from Tecan Austria GmbH (Untersbergstr.1A, A-5082 Groedig, Austria, Serial No.: 2310004278), and CO_2 Incubator (Serial No.: K1223K7556) was from Lishen Scientific Equipment Co. Ltd.

2.2 Measurement of nitric oxide (NO) production

NO production was quantified by measuring nitrite accumulation in the cell culture supernatants using Griese reagent [1]. Briefly, RAW 264.7 macrophages (6×10^3 cells/mL) were seeded in 96-well plates and pretreated with test compounds for 1 h prior to LPS stimulation (1μg/mL). Culture supernatants were then mixed with Griese reagent (Beyotime Biotechnology, China). A standard curve was generated using $NaNO_2$, and absorbance was measured at 540 nm. N^G-monomethyl-l-arginine monoacetate (L-NMMA) served as the positive control.

2.3 Cytotoxicity Assay

The cytotoxic effects of the compounds were evaluated against human colon cancer cells (HCT-116) and human liver cancer cells (Hep3B) using the MTT assay. HCT-116 and Hep3B cells were seeded into 96-well culture plates at a density of 5×10^3 cells per well and incubated for 24 h. Various concentrations of test compounds (**1**−**4**) were added to the cells. After 48 h, 20 μL MTT (5 mg/mL) was added to each well, and followed by incubation for 4 h at 37 °C. Then, the supernatant was aspirated and DMSO was added by continuous shaking. Absorbance was measured using a microplate reader at 570 nm.[2]

References

[1] R. Tao, P. Tang, J. Gao, J. Li, Y. Sun, J. Luo, Y. Li, *Phytomedicine*, 2022, **98,** 153952.

[2] Y. Sun, Y. Li, L. Cui, Q. Li, S. Wang, Z. Chen, L.-Y. Kong, J. Luo*, J. Agric. Food Chem.,* 2023, **71**, 14000−14012.

3. Computational details

3.1 NMR calculations

The initial conformational analysis of the compound **2** was executed by employing Monte Carlo searching algorithm via the MMFF94 molecular mechanics force field [1], with the aid of the SPARTAN'16 program package, leading to afford a panel of relatively favored conformations in an energy range of 3 kcal/mol above the global minimum. The force field minimum energy conformers thus obtained were subsequently optimized by applying the density functional theory (DFT) with the B3LYP/6-31G(d) level in vacuum, implemented in the Gaussian 09 software package[2]. Harmonic vibrational frequencies were also performed to confirm no imaginary frequencies of the finally optimized conformers. Gauge-Independent Atomic Orbital (GIAO) calculations of NMR chemical shifts were accomplished by DFT at the mPW1PW91/6-311+g (d, p) level in Chloroform with the PCM solvent model in Gaussian 09 software. NMR chemical shifts of TMS were calculated in the same level and used as the references. Regression analysis of calculated versus experimental NMR chemical shifts of **2** was carried out. Linear correlation coefficients (R^2) and Root-mean-square deviation (RMSD) were calculated for the evaluation of the results.

After Boltzmann weighing of the predicted chemical shift of each isomers, the DP4+ parameters were calculated using the excel file provided by Ariel M. Sarotti [3].

3.2 ECD Computational details

The initial conformational analysis of the compounds **2** and **3** were executed by employing Monte Carlo searching algorithm via the MMFF94 molecular mechanics force field [1], with the aid of the SPARTAN'16 program package, leading to afford a panel of relatively favored conformations in an energy range of 3 kcal/mol above the global minimum. The force field minimum energy conformers thus obtained were subsequently optimized by applying the density functional theory (DFT) with the B3LYP/6-31G(d) level in vacuum, implemented in the Gaussian 09 software package [2]. Harmonic vibrational frequencies were also performed to confirm no imaginary frequencies of the finally optimized conformers. These predominant conformers were subjected to theoretical calculation of ECD by utilizing Time-dependent

density functional theory (TDDFT) calculations at the B3LYP/6-311g (2d, p) level in MeOH using the Polarizable Continuum Model (PCM) solvent model. The energies, oscillator strengths, and rotational strengths of each conformers were carried out with Gaussian 09 software package. The oretical calculations of ECD spectra for each conformer were then approximated by the Gaussian distribution. The final ECD spectrum of the individual conformers was summed up on the basis of Boltzmann-weighed population contribution by the SpecDisv1.64 [3].

Table S1. Energy analyses of conformers (5*S*, 10*S*, 13*S*, 14*R*)-**2A**a-f

NO.	3D comformers	Free energy		
		E (Hartree)	ΔE (Kcal/mol)	Boltzmann distribution
2Aa	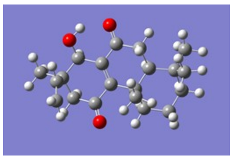	-1003.505457	0.0000	69.03%
2Ab	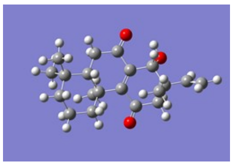	-1003.504053	0.8809	15.59%
2Ac	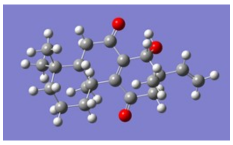	-1003.503482	1.2392	8.51%
2Ad	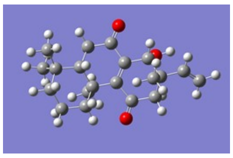	-1003.503044	1.5143	5.35%

Table S2. Energy analyses of conformers (5*S*, 10*S*, 13*R*, 14*S*)-**2B**a-c

NO.	3D comformers	Free energy		
		E (Hartree)	ΔE (Kcal/mol)	Boltzmann distribution
1Ba	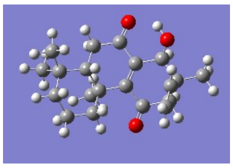	-1003.507578	0.0000	96.35%

1Bb	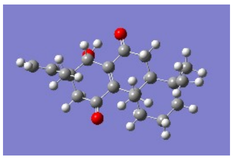	-1003.503862	2.3318	1.88%
1Bc	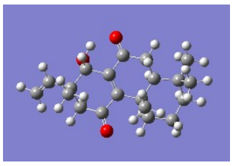	-1003.503476	2.5470	1.25%

Table S3. Energy analyses of conformers (5*S*, 7*S*, 9*R*, 10*S*, 12*R*, 13*R*)-**3**a-f

NO.	3D comformers	Free energy		
		E (Hartree)	ΔE (Kcal/mol)	Boltzmann distribution
3a	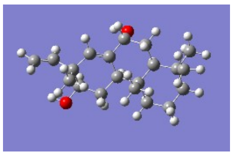	-930.6974555	0	64.59%
3b	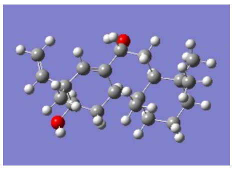	-930.6955441	1.1994	8.52%
3c	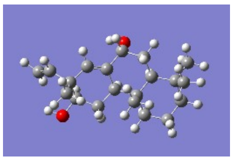	-930.6954315	1.2701	7.56%
3d	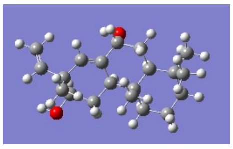	-930.6954082	1.2847	7.38%
3e	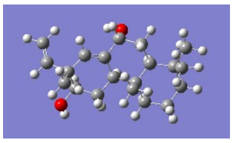	-930.6950827	1.4889	5.22%
3f	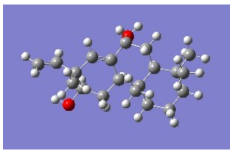	-930.6948818	1.6151	4.22%
3g	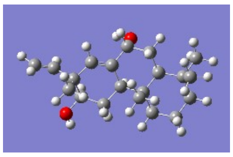	-930.6941067	2.1014	1.86%

References

[1] Halgren TA. *J Comput Chem*, 1999, **20**, 730-748.

[2] Frisch MJ, Trucks GW, Schlegel H B, Scuseria GE, Robb MA, Cheeseman JR, Scalmani G, Barone V, Mennucci B, Petersson GA, Nakatsuji H, Caricato M, Li X, Hratchian H P, Izmaylov AF, Bloino J, Zheng G, Sonnenberg JL, Hada M, Ehara M, Toyota K, Fukuda R, Hasegawa J, Ishida M, Nakajima T, Honda Y, Kitao O, Nakai H, Vreven T, Montgomery Jr JA, Peralta JE, Ogliaro F, Bearpark M, Heyd JJ, Brothers E, Kudin KN, Staroverov VN, Kobayashi R, Normand J, Raghavachari K, Rendell A, Burant JC, Iyengar SS, Tomasi J, Cossi M, Rega N, Millam JM, Klene M, Knox JE, Cross J B, Bakken V, Adamo C, Jaramillo J, Gomperts R, Stratmann RE, Yazyev O, Austin AJ, Cammi R, Pomelli C, Ochterski JW, Martin RL, Morokuma K, Zakrzewski VG, Voth GA, Salvador P, Dannenberg JJ, Dapprich S, Daniels AD, Farkas Ö, Foresman JB, Ortiz JV, Cioslowski J, Fox DJ, Gaussian 09, Rev. C 01, Gaussian, Inc., Wallingford CT, 2009.

[3] Bruhn T, Schaumloffel A, Hemberger Y, Bringmann G. *Chirality*, 2013, **25**, 243-24.

4. Original spectra of compound

NMR, MS, UV and CD spectra of compound 1

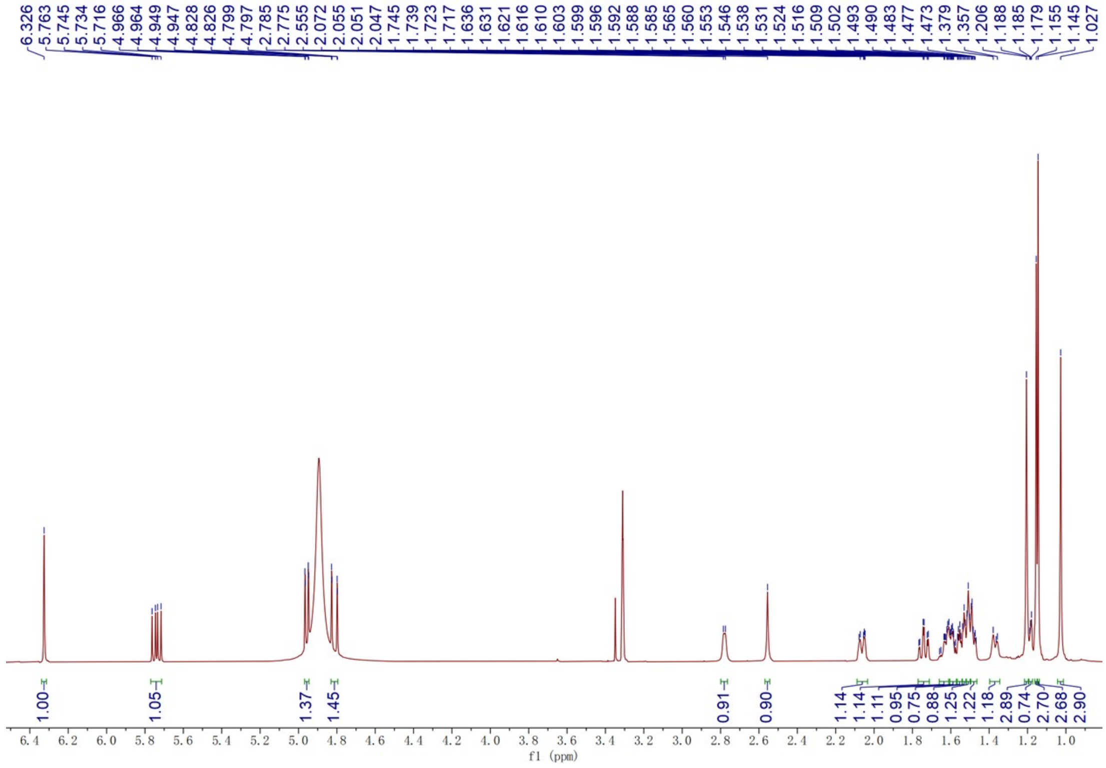

Fig. S4 ^{1}H NMR spectrum of compound **1** in CD_3OD

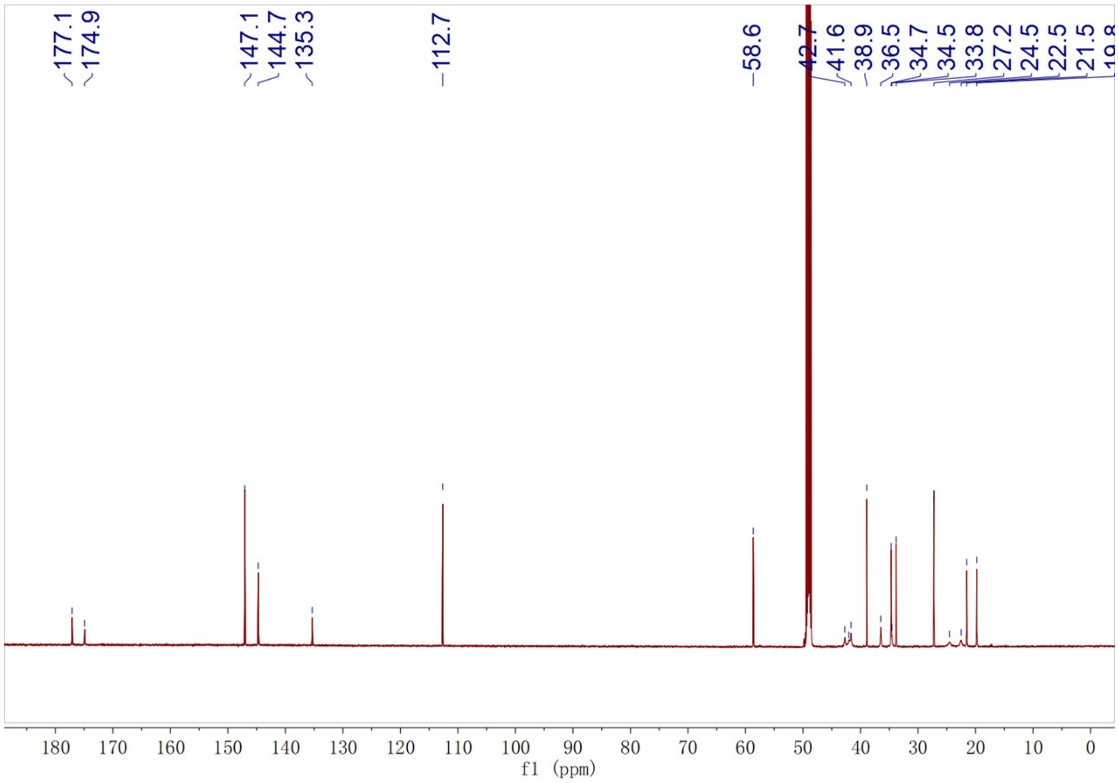

Fig. S5 ^{13}C NMR spectrum of compound **1** in CD_3OD

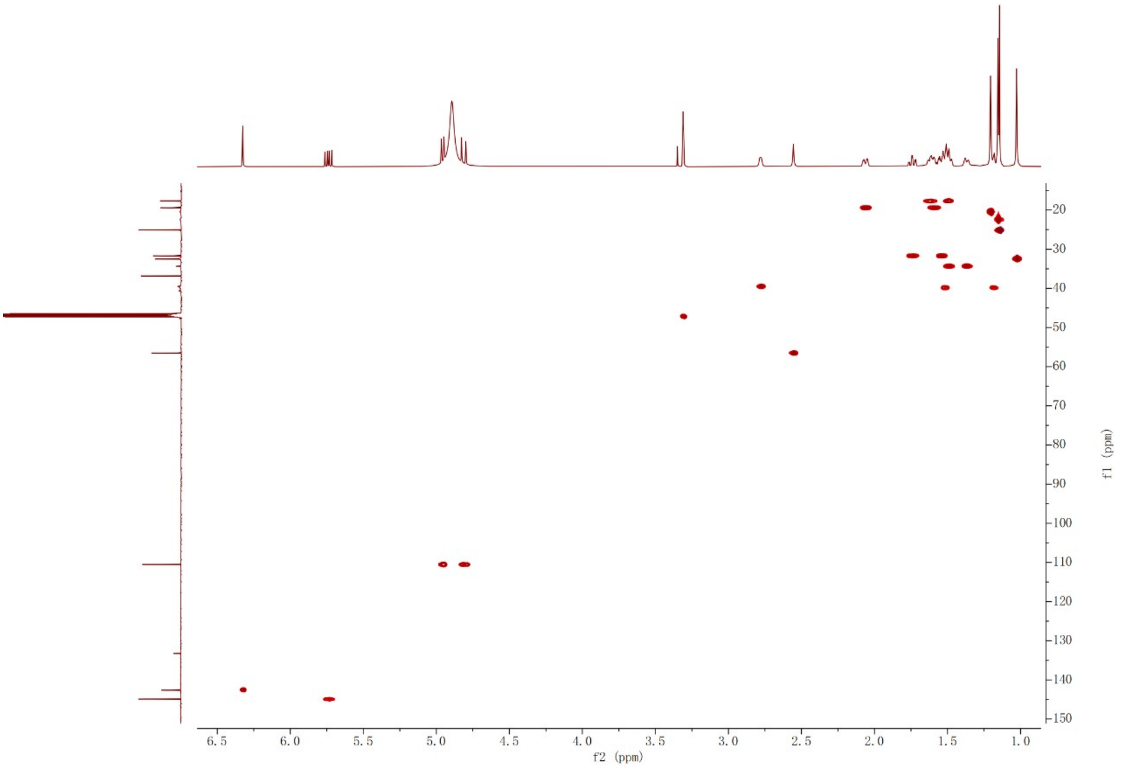

Fig. S6 HSQC spectrum of compound **1** in CD_3OD

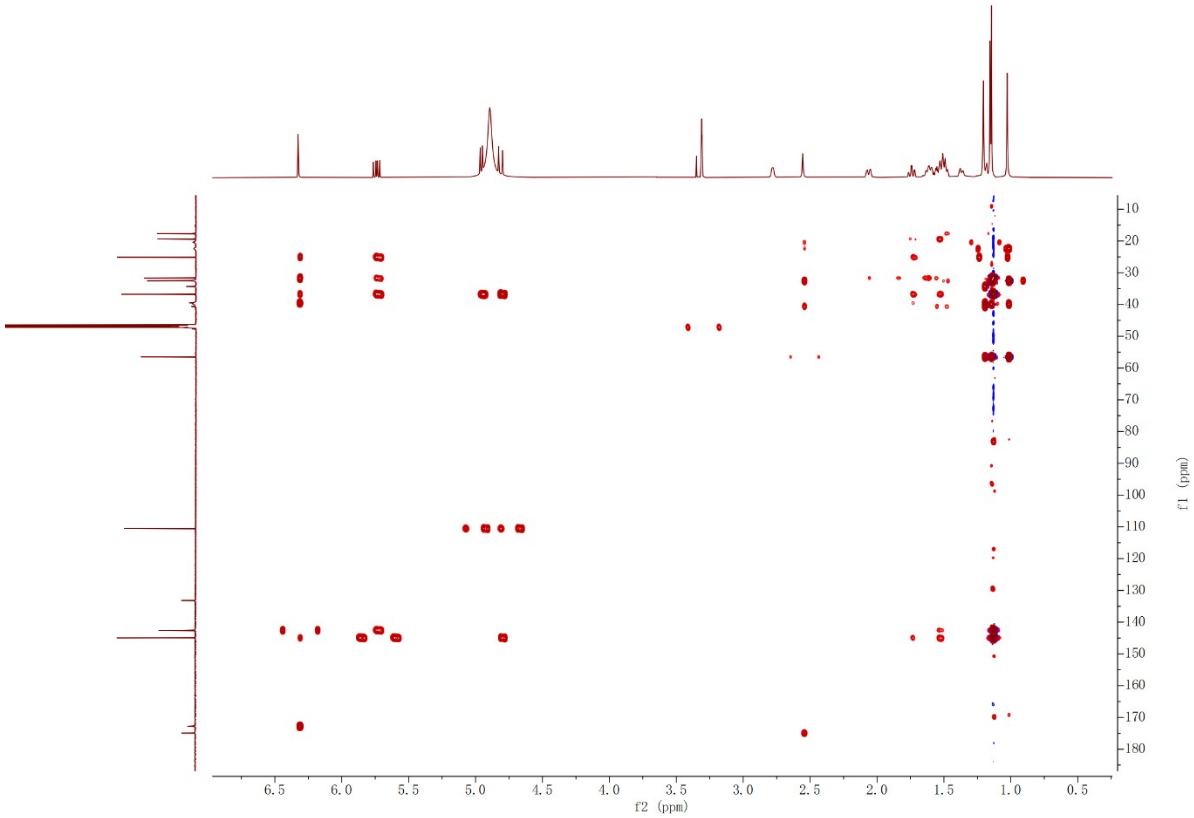

Fig. S7 HMBC spectrum of compound **1** in CD_3OD

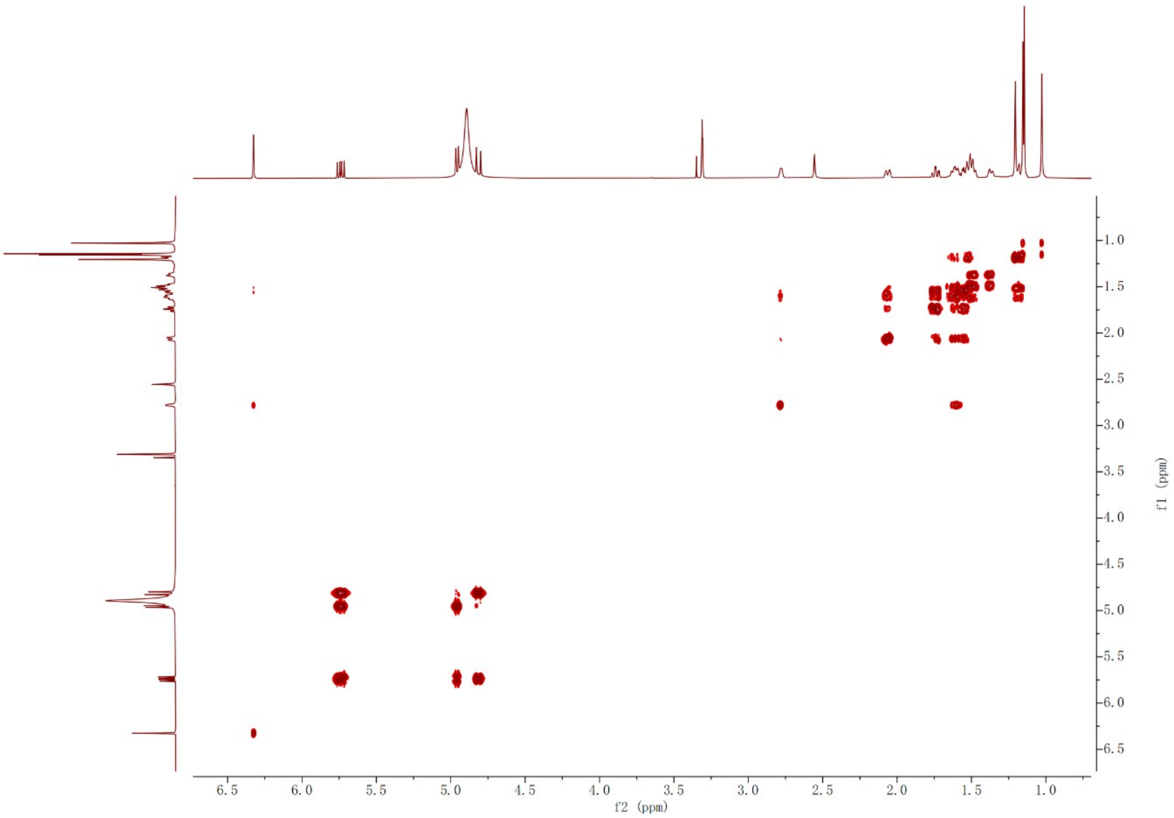

Fig. S8 ^{1}H-^{1}H COSY spectrum of compound **1** in CD_3OD

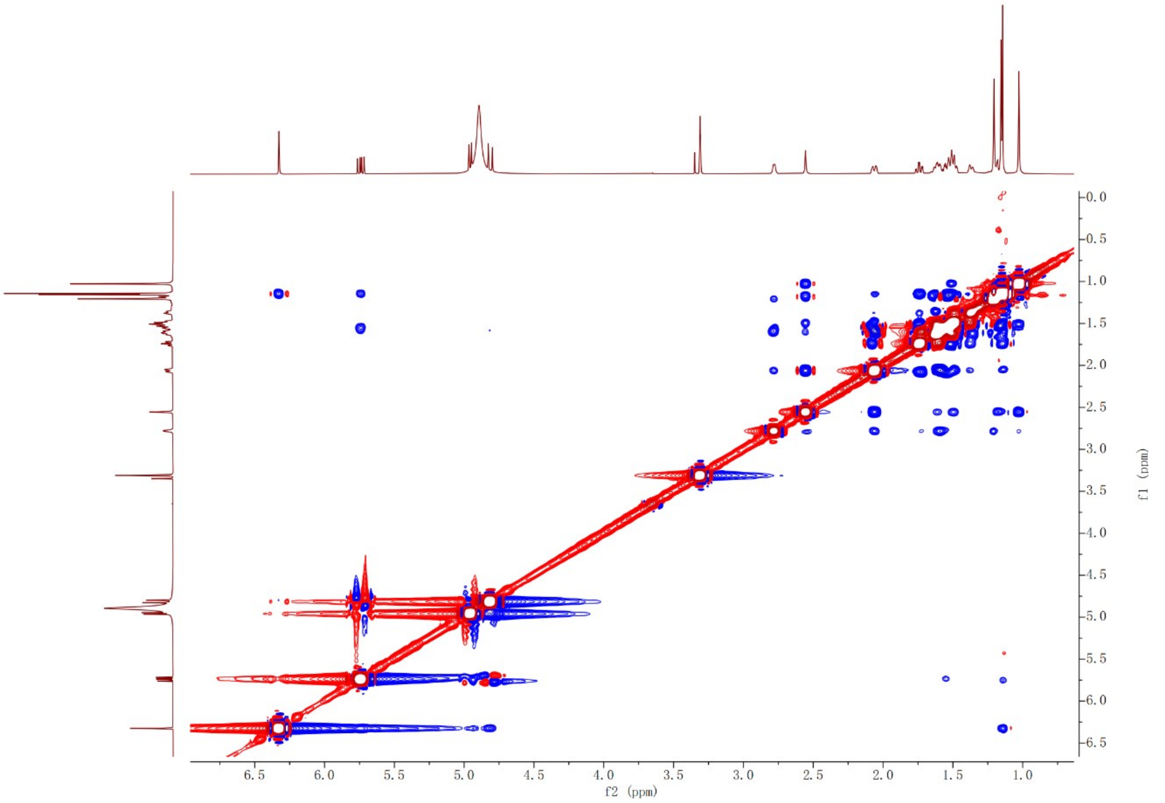

Fig. S9 ROESY spectrum of compound **1** in CD_3OD

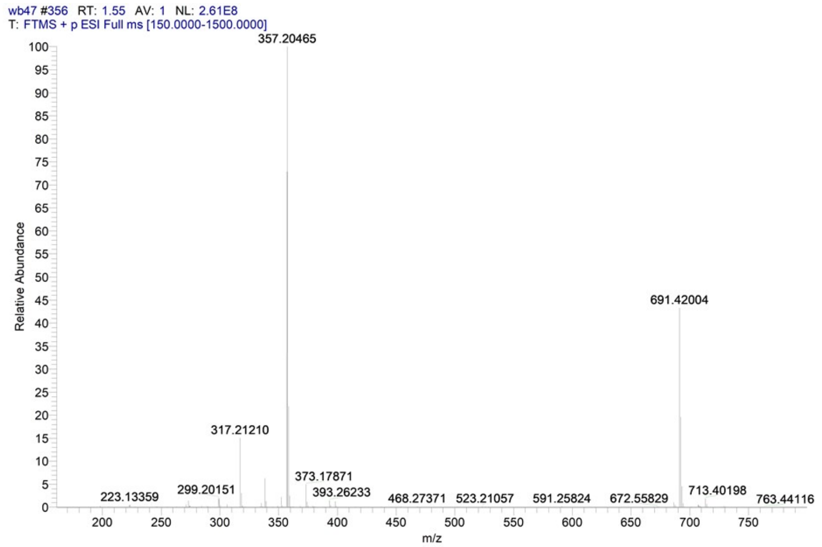

m/z	Theo.Mass	Delta(ppm)	RDB equiv	Composition	
357.20465	357.20363	2.86	5.50	$C_{20}H_{30}O_4$	$[M+Na]^+$

Fig. S10 HR-ESI-MS spectrum of compound **1**

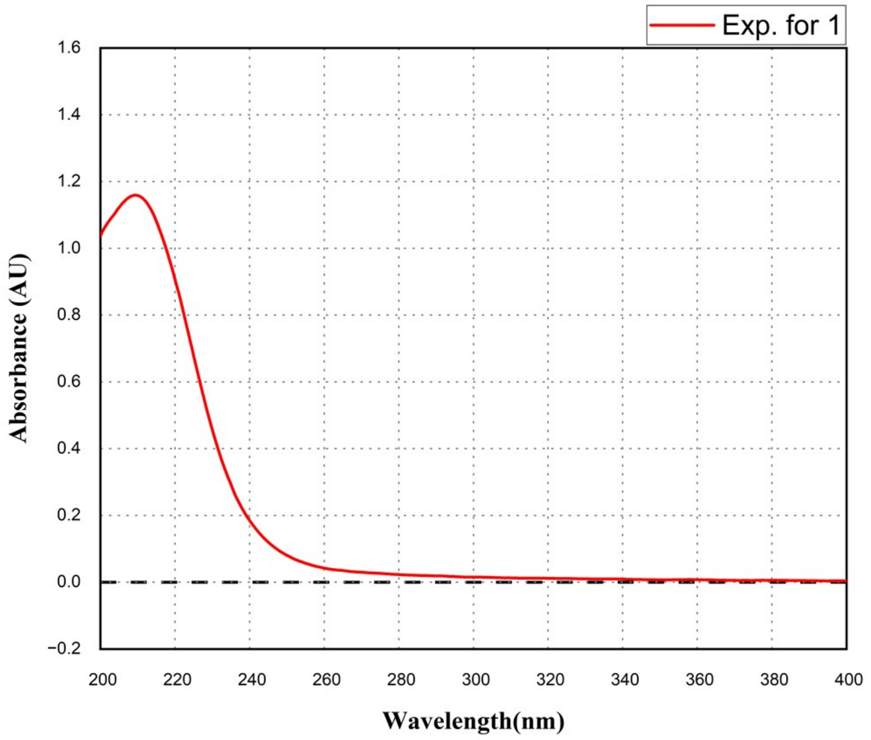

Fig. S11 UV (MeOH) spectrum of **1**

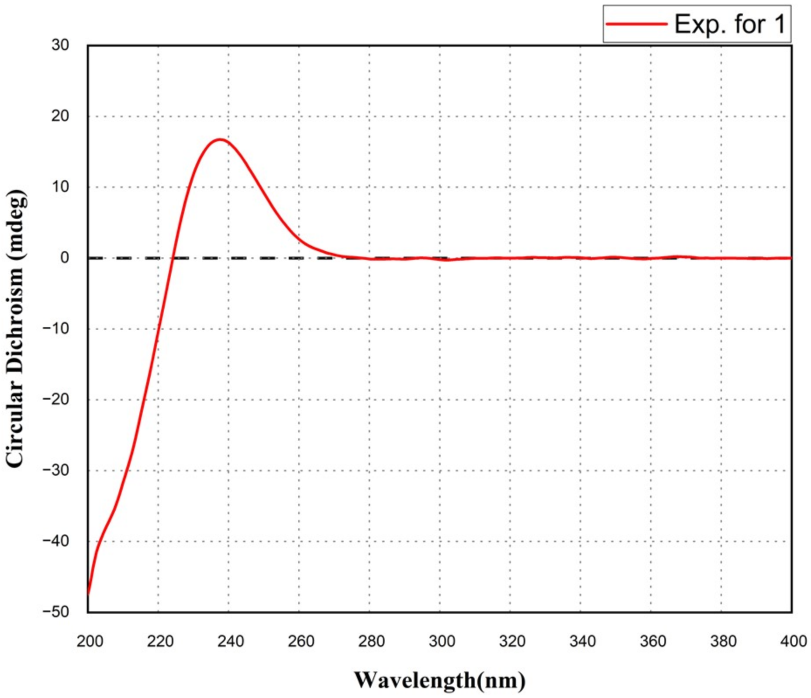

Fig. S12 CD (MeOH) spectrum of **1**

NMR, MS, UV and CD spectra of compound 2

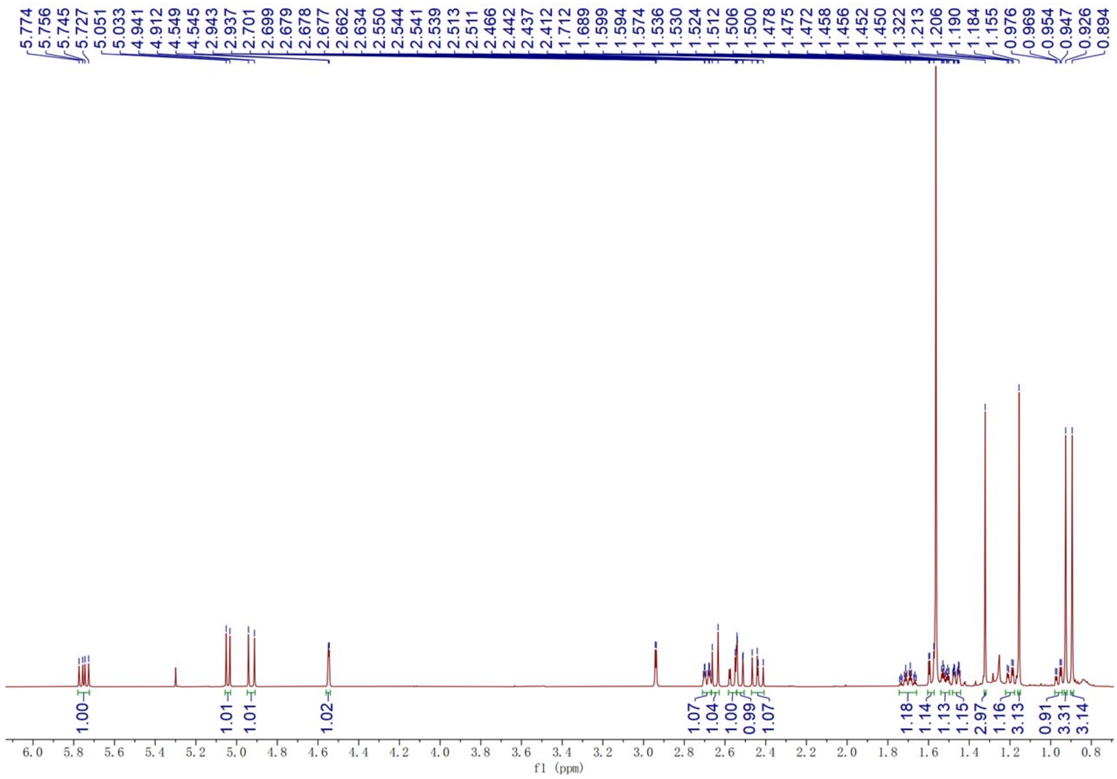

Fig. S13 ^{1}H NMR spectrum of compound **2** in $CDCl_3$

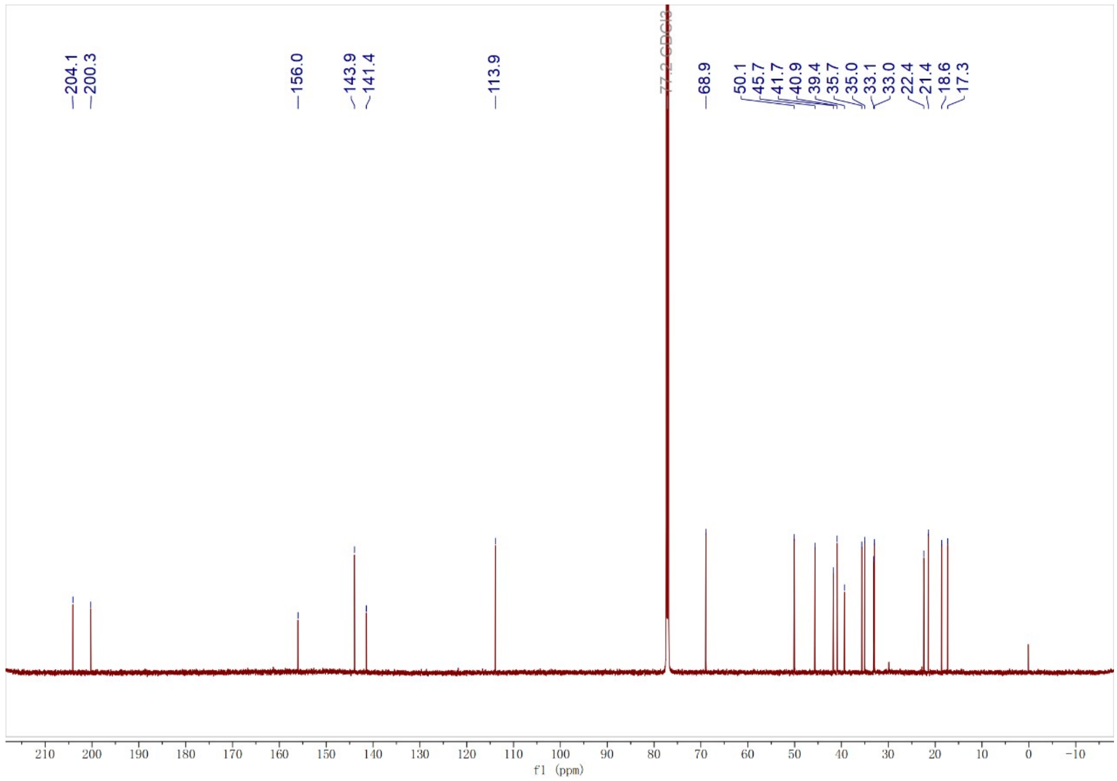

Fig. S14 ^{13}C NMR spectrum of compound **2** in $CDCl_3$

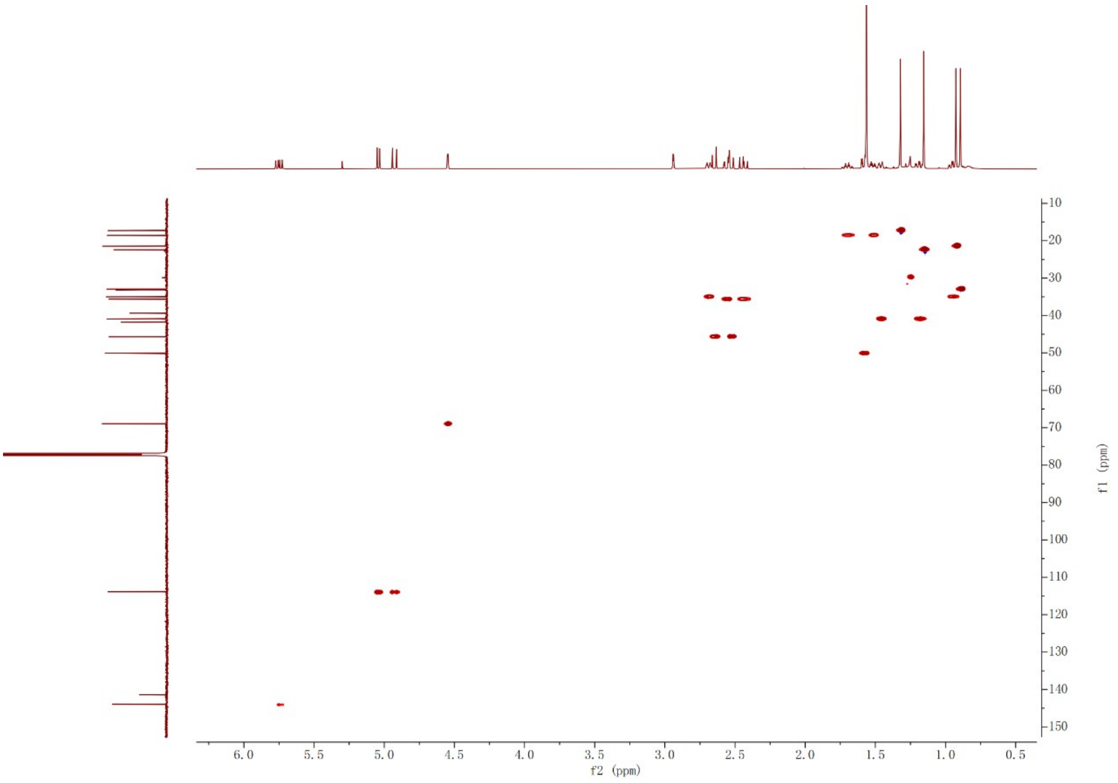

Fig. S15 HSQC spectrum of compound **2** in $CDCl_3$

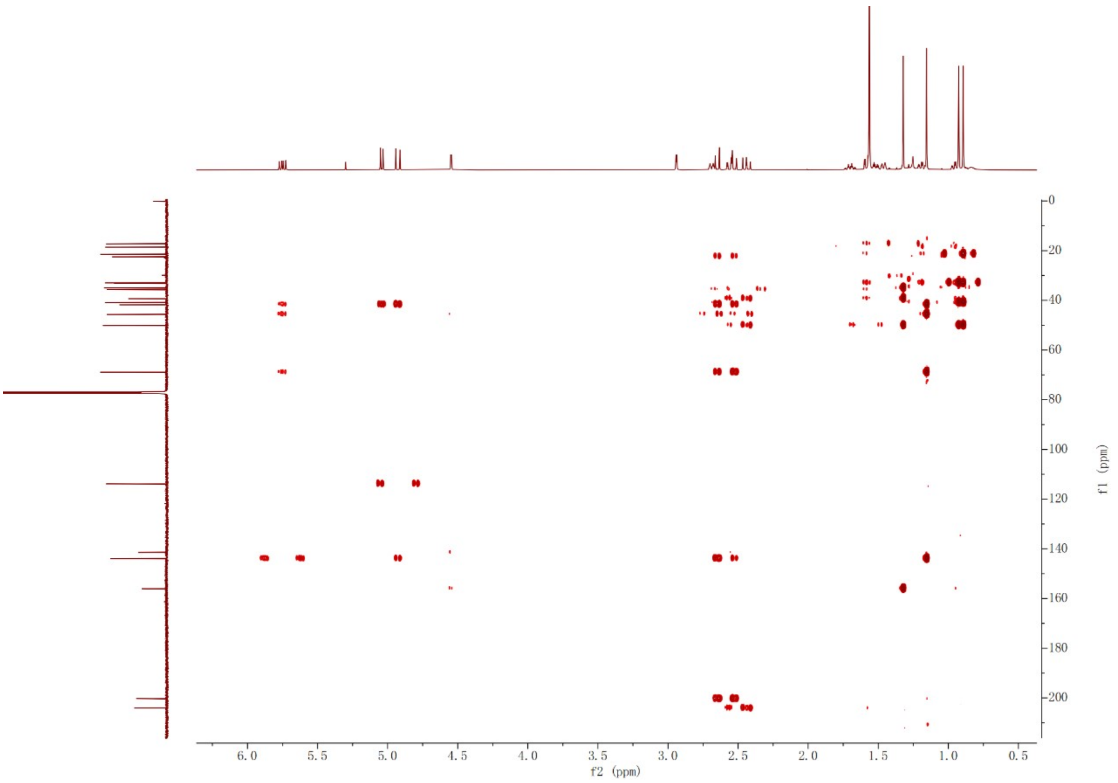

Fig. S16 HMBC spectrum of compound **2** in $CDCl_3$

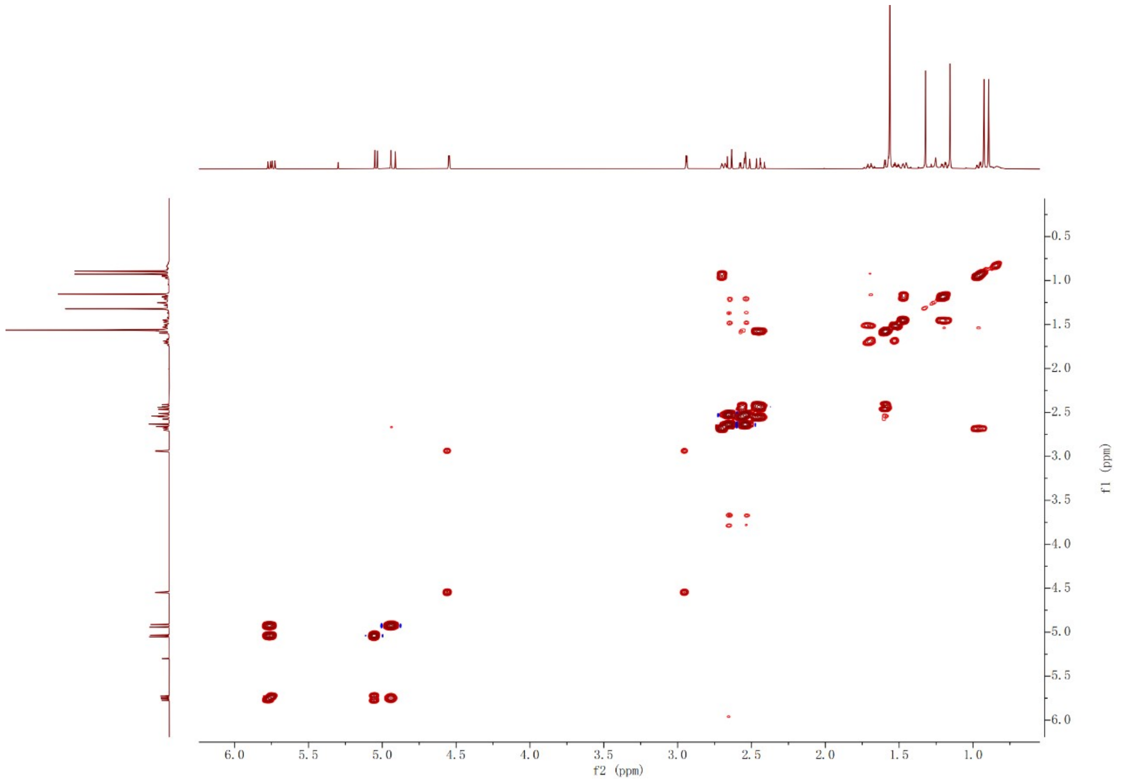

Fig. S17 ^{1}H-^{1}H COSY spectrum of compound **2** in $CDCl_3$

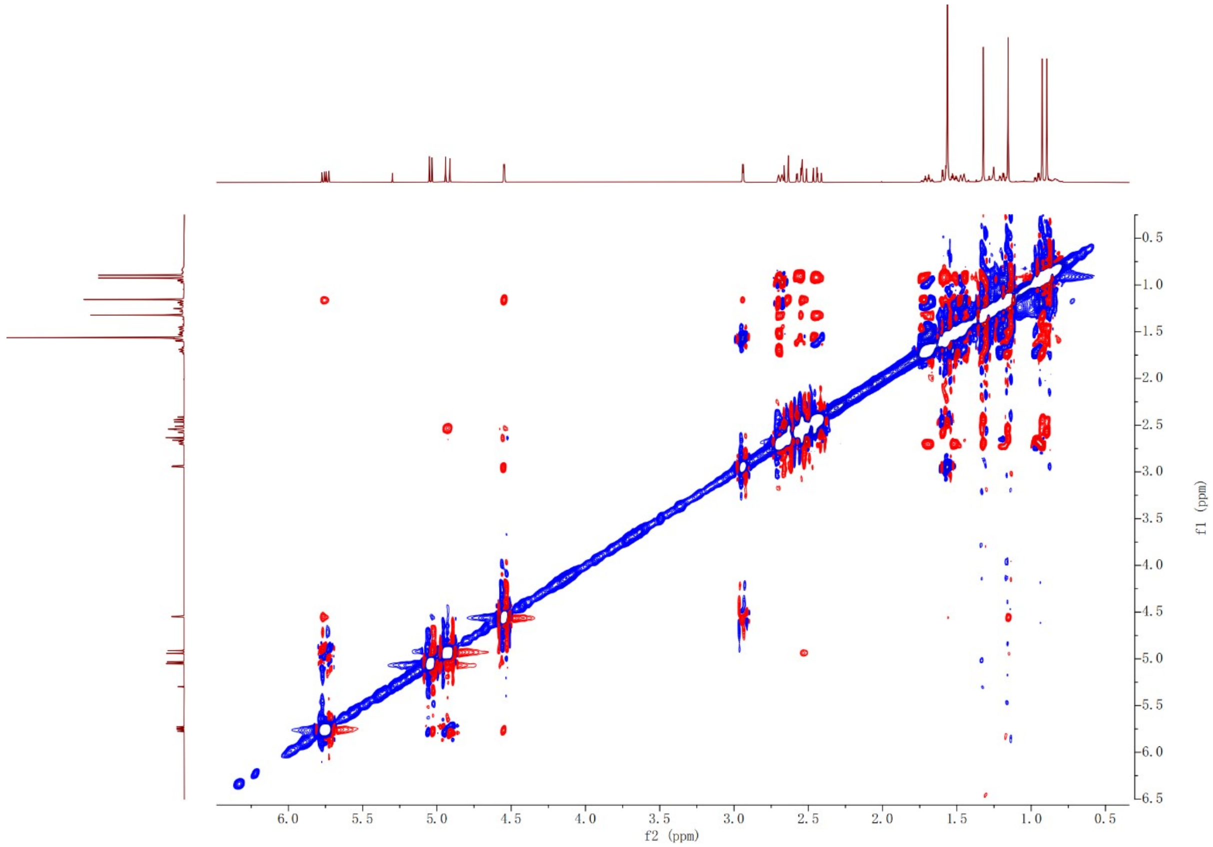

Fig. S18 ROESY spectrum of compound **2** in $CDCl_3$

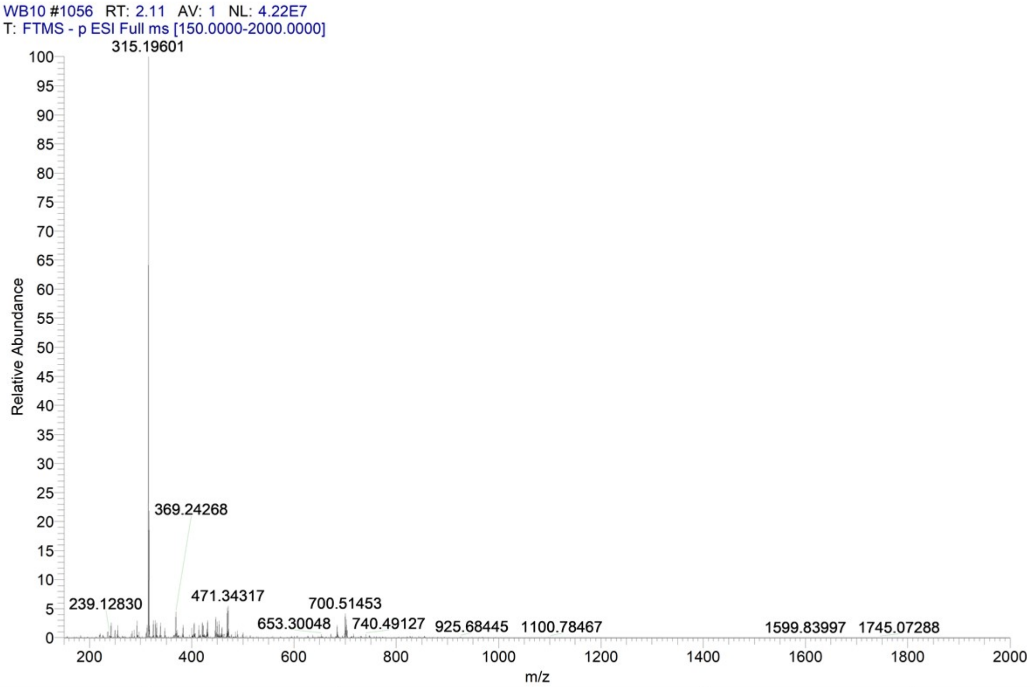

m/z	Theo.Mass	Delta(ppm)	RDB equiv.	Composition	
315.19601	315.19547	1.70	7.50	$C_{20}H_{28}O_3$	$[M-H]^-$

Fig. S19 HRESIMS spectrum of compound **2**

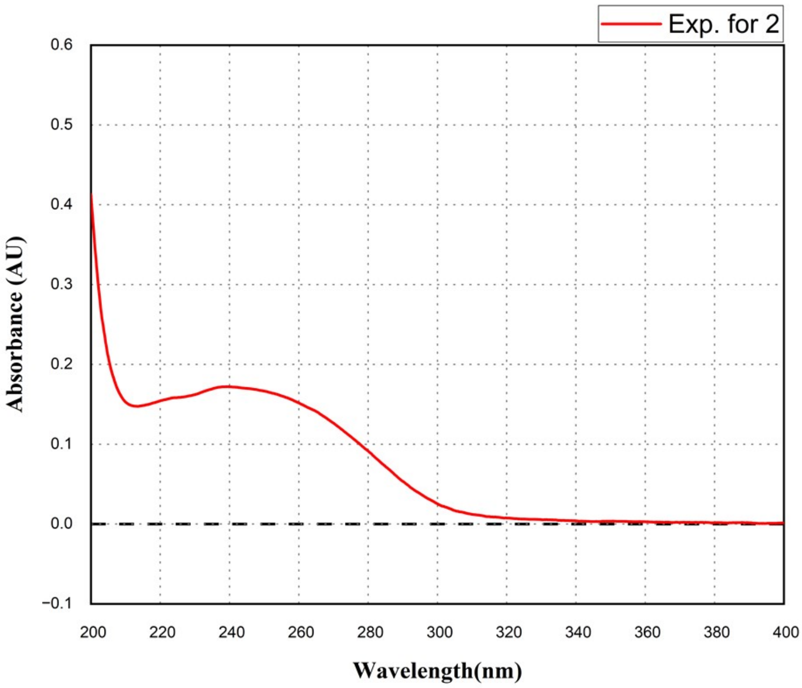

Fig. S20 UV (MeOH) spectrum of **2**

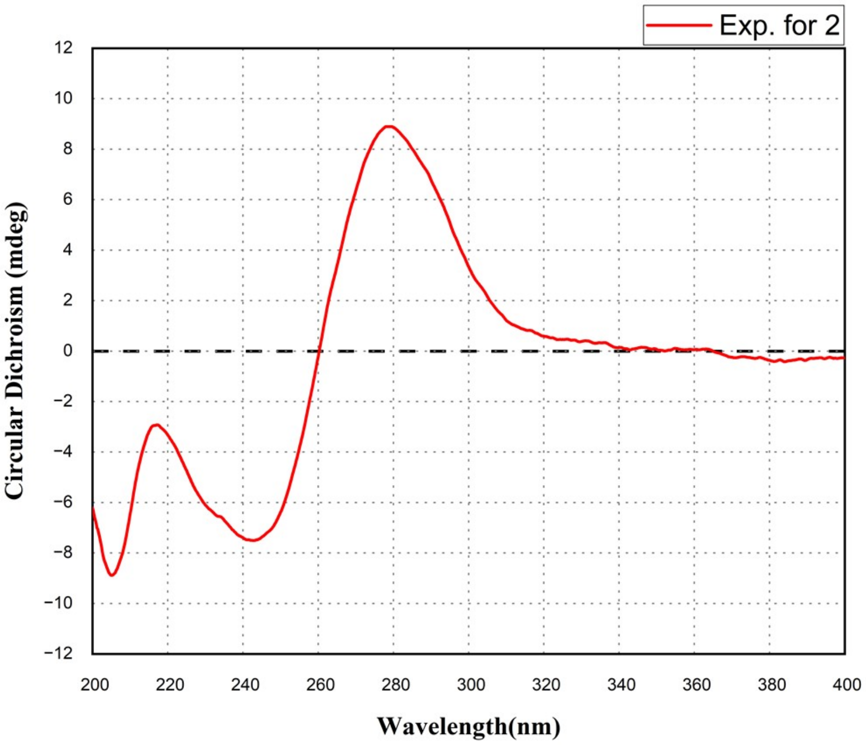

Fig. S21 CD (MeOH) spectrum of **2**

NMR, MS, UV and CD spectra of compound 3

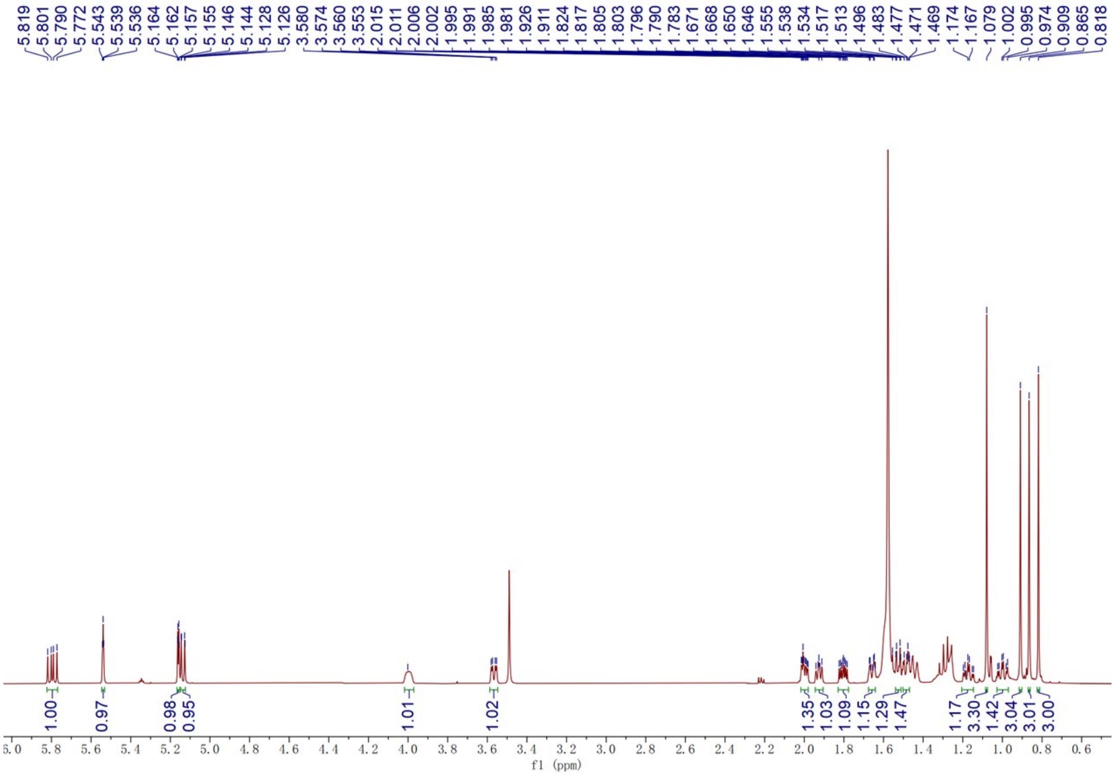

Fig. S22 ^{1}H NMR spectrum of compound **3** in $CDCl_3$

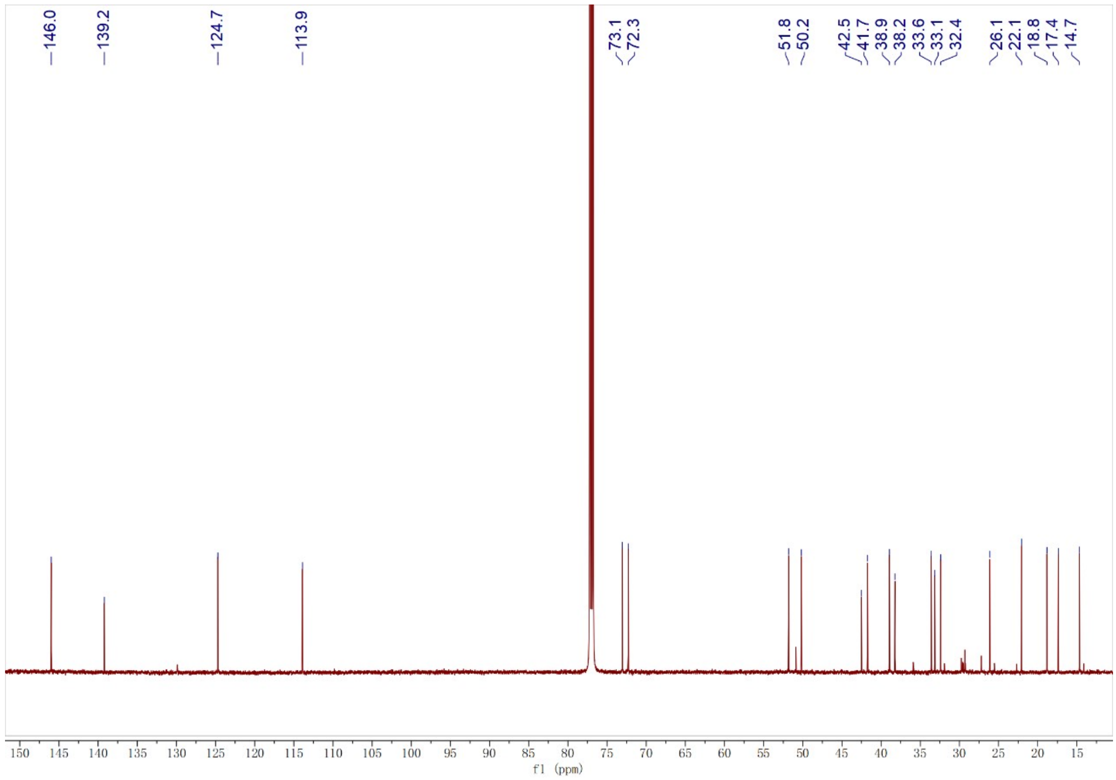

Fig. S23 ^{13}C NMR spectrum of compound **3** in $CDCl_3$

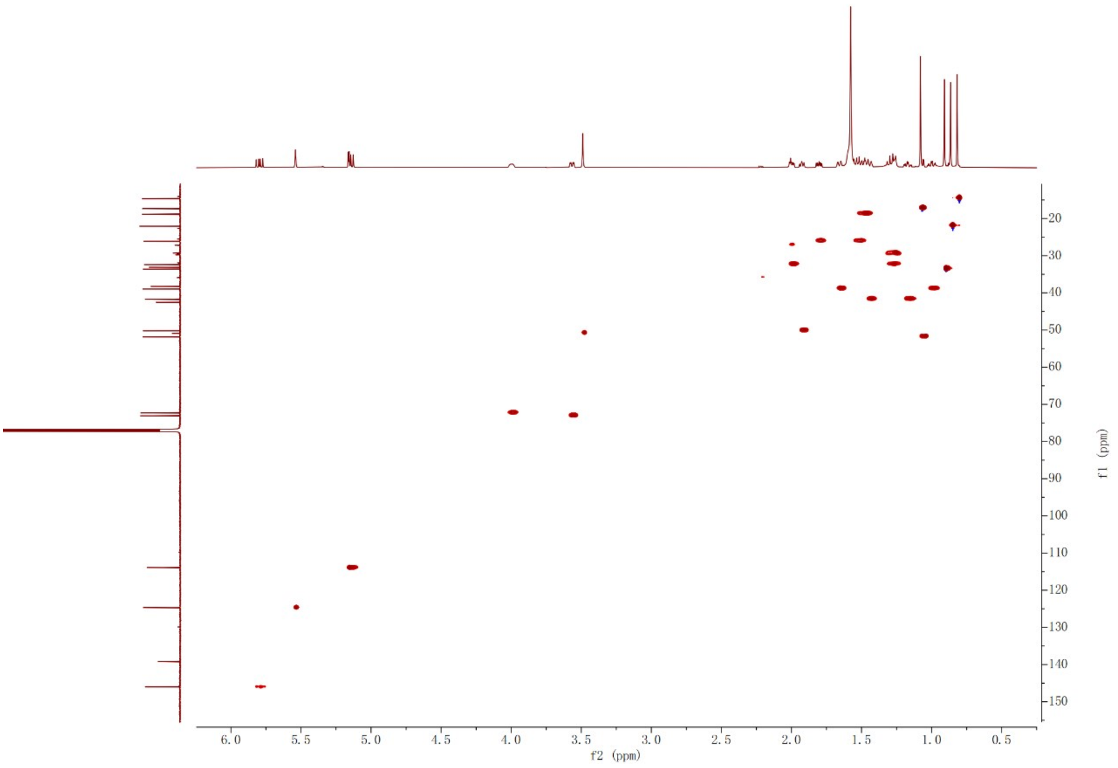

Fig. S24 HSQC spectrum of compound **3** in $CDCl_3$

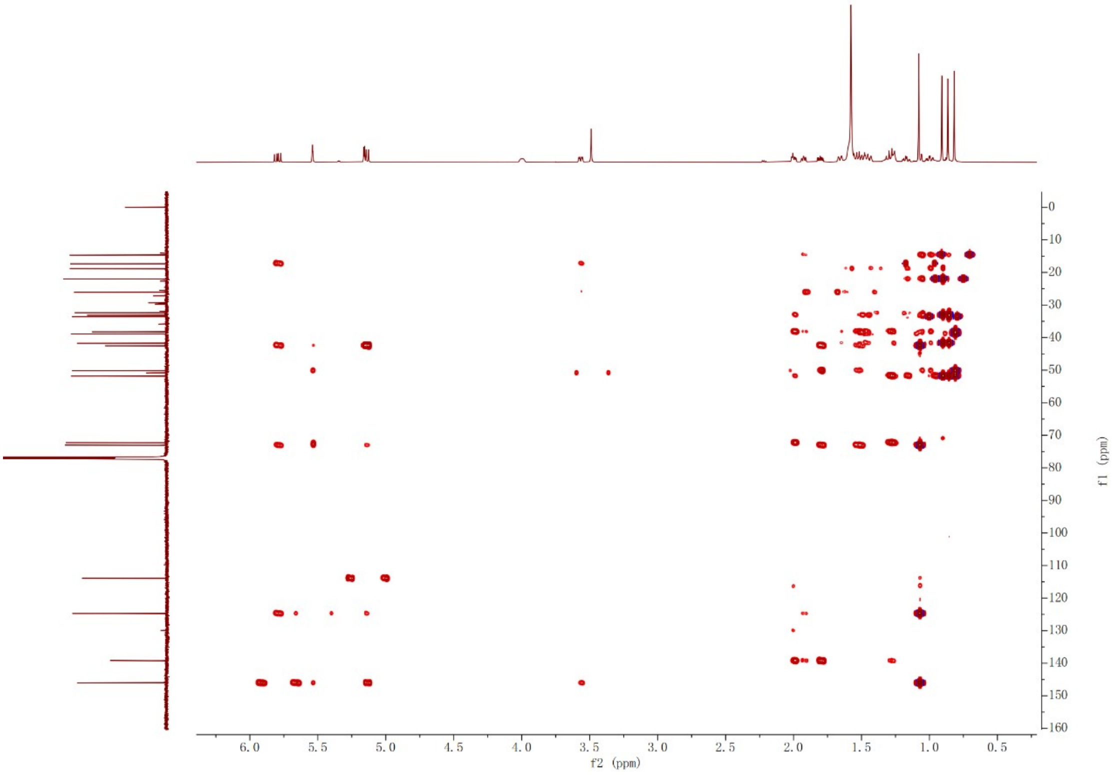

Fig. S25 HMBC spectrum of compound **3** in $CDCl_3$

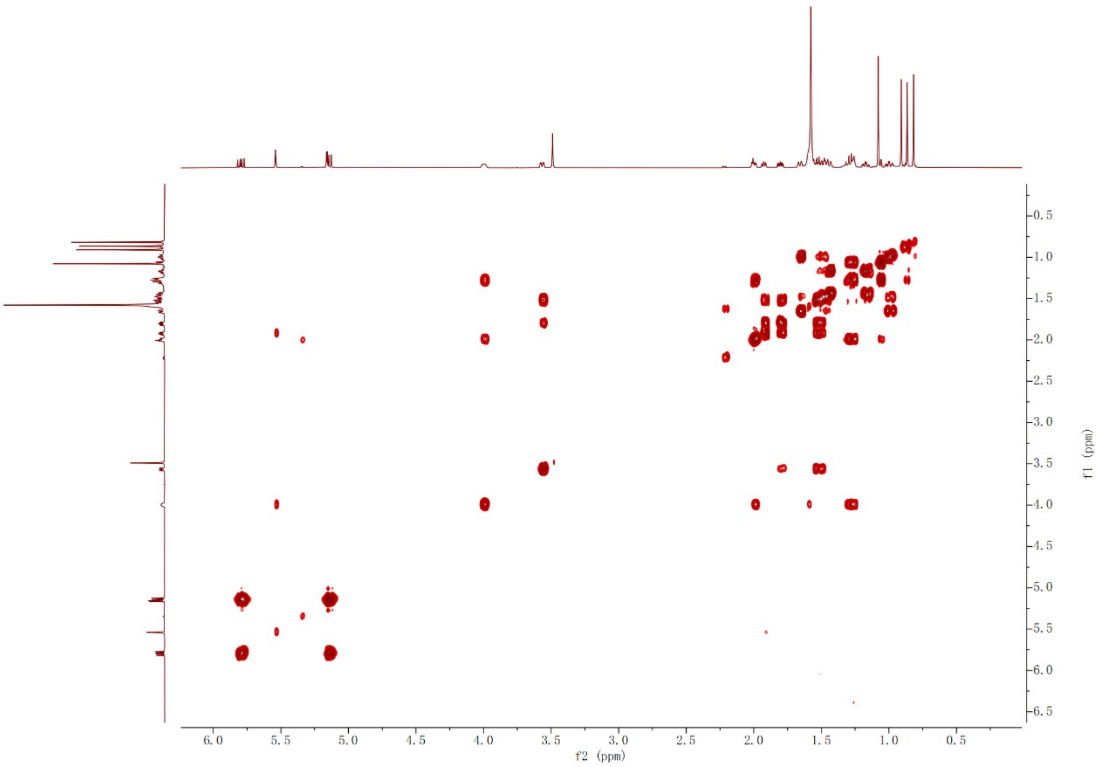

Fig. S26 ^{1}H-^{1}H COSY spectrum of compound **3** in $CDCl_3$

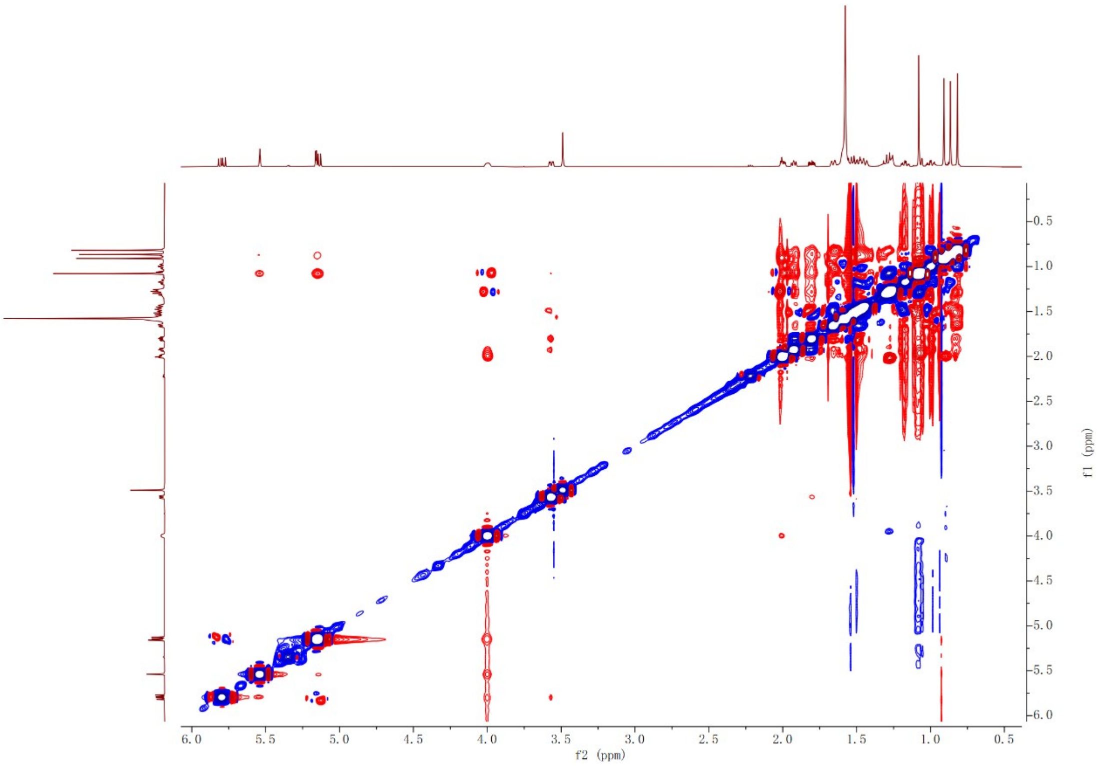

Fig. S27 ROESY spectrum of compound **3** in $CDCl_3$

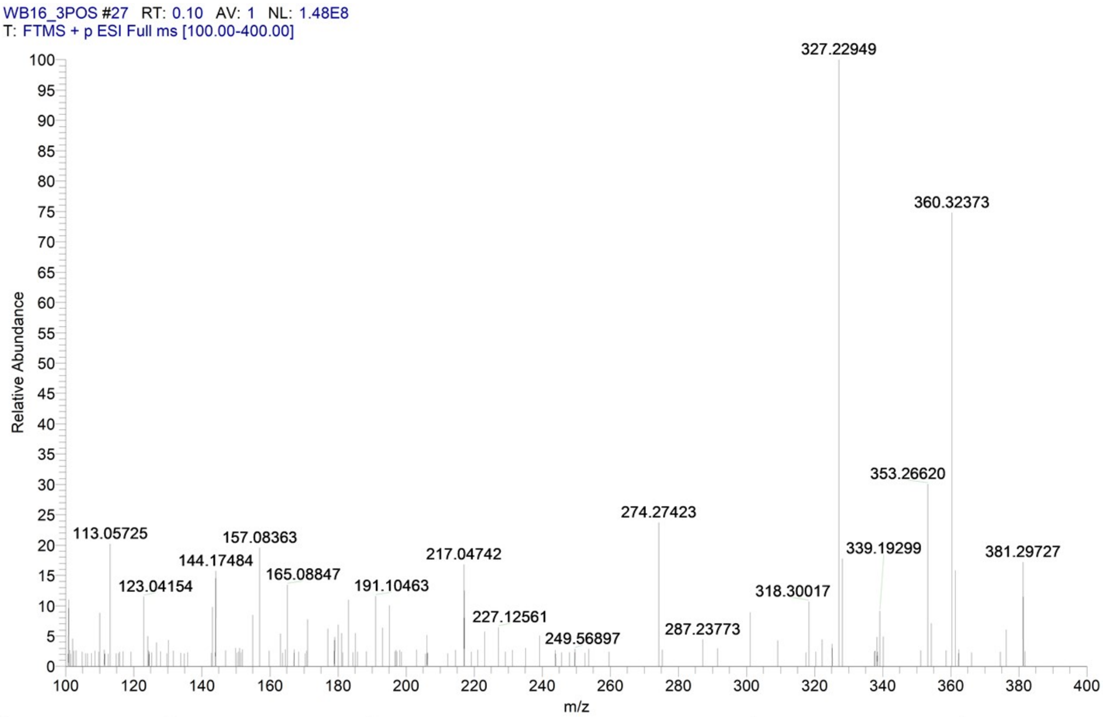

m/z	Theo.Mass	Delta(ppm)	RDB equiv	Composition	
327.22949	327.22945	0.12	4.50	$C_{20}H_{32}O_2$	$[M+Na]^+$

Fig. S28 HRESIMS spectrum of compound **3**

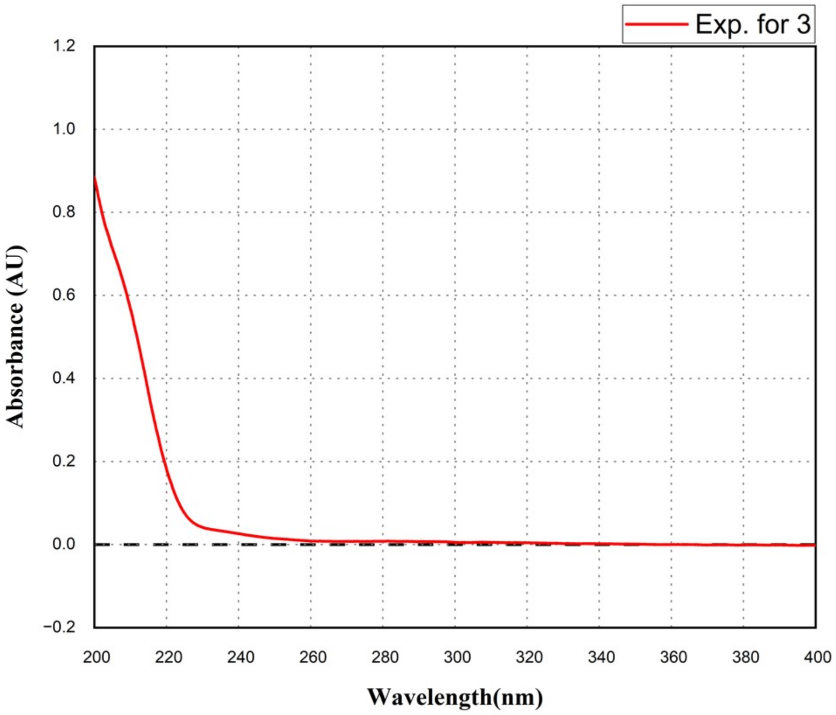

Fig. S29 UV (MeOH) spectrum of **3**

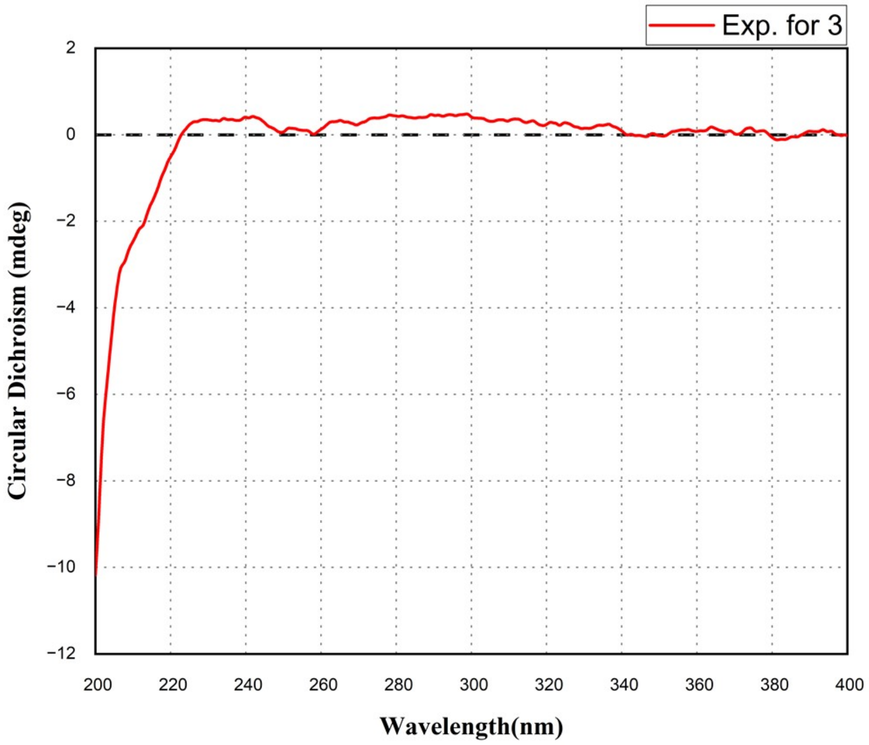

Fig. S30 CD (MeOH) spectrum of **3**

NMR spectra of compound 4

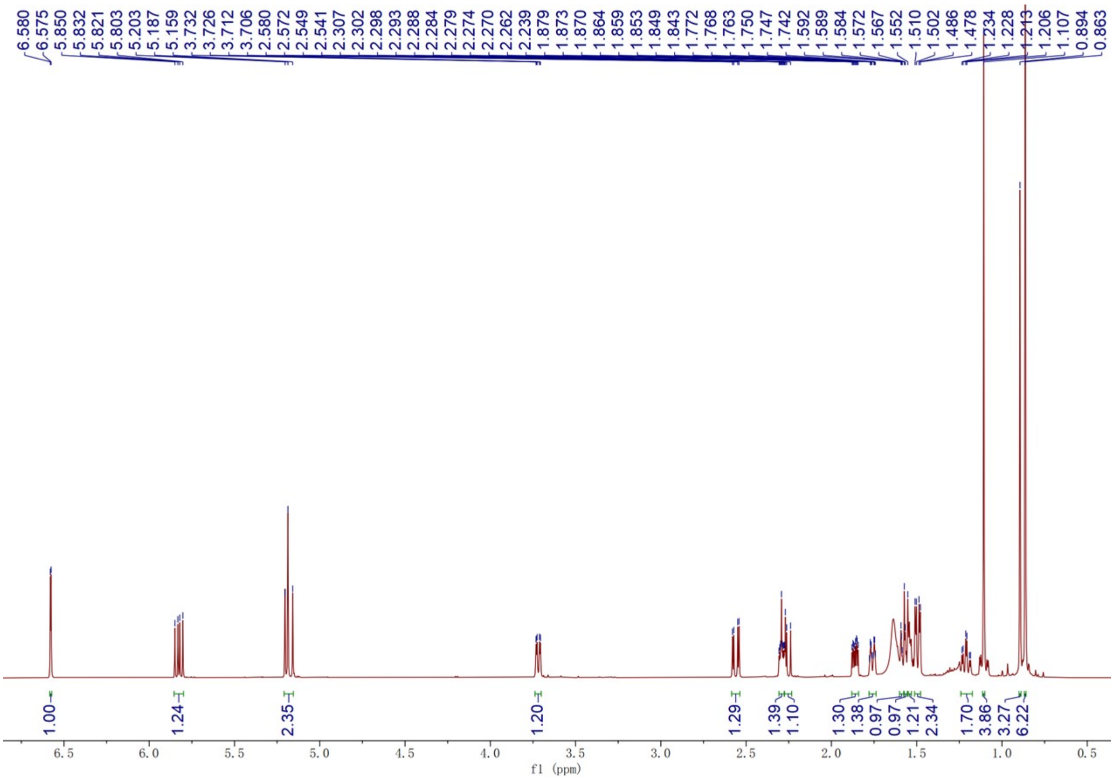

Fig. S31 ^{1}H NMR spectrum of compound **4** in $CDCl_3$

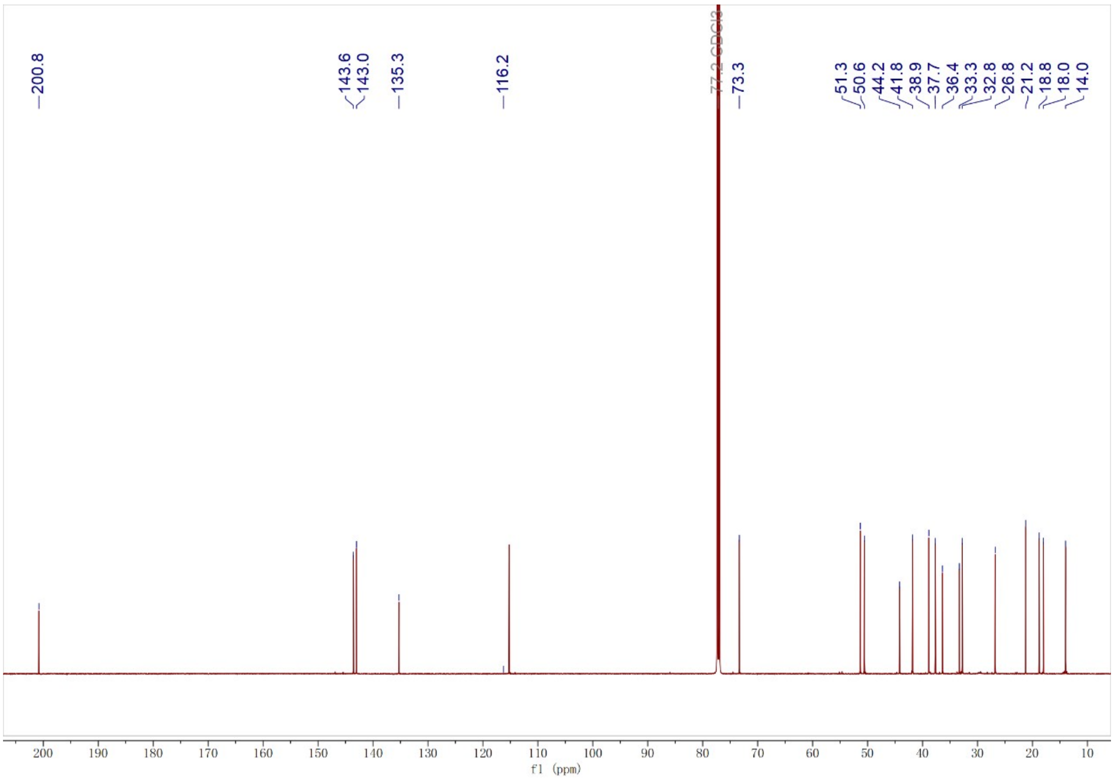

Fig. S32 ^{13}C NMR spectrum of compound **4** in $CDCl_3$
